# DE-kupl: exhaustive capture of biological variation in RNA-seq data through *k*-mer decomposition

**DOI:** 10.1186/s13059-017-1372-2

**Published:** 2017-12-28

**Authors:** Jérôme Audoux, Nicolas Philippe, Rayan Chikhi, Mikaël Salson, Mélina Gallopin, Marc Gabriel, Jérémy Le Coz, Emilie Drouineau, Thérèse Commes, Daniel Gautheret

**Affiliations:** 1 INSERM U1183 IRMB, Université de Montpellier, Hopital St Eloi, 80 avenue Augustin Fliche, Montpellier, 34295 France; 2 Institut de Biologie Computationnelle, Université Montpellier, Montpellier, France; 3 SeqOne, IRMB, CHRU de Montpellier, Hopital St Eloi, Montpellier, France; 4 Univ. Lille, CNRS, Inria, UMR 9189 - CRIStAL - F-59000, Lille, France; 5 Institute for Integrative Biology of the Cell, CEA, CNRS, Université Paris-Sud, Université Paris Saclay, Gif sur Yvette, France; 6 Institut de Cancérologie Gustave Roussy Cancer Campus (GRCC), AMMICA, INSERM US23/CNRS UMS3655, Villejuif, France

## Abstract

We introduce a *k*-mer-based computational protocol, DE-kupl, for capturing local RNA variation in a set of RNA-seq libraries, independently of a reference genome or transcriptome. DE-kupl extracts all *k*-mers with differential abundance directly from the raw data files. This enables the retrieval of virtually all variation present in an RNA-seq data set. This variation is subsequently assigned to biological events or entities such as differential long non-coding RNAs, splice and polyadenylation variants, introns, repeats, editing or mutation events, and exogenous RNA. Applying DE-kupl to human RNA-seq data sets identified multiple types of novel events, reproducibly across independent RNA-seq experiments.

**Electronic supplementary material:**

The online version of this article (doi:10.1186/s13059-017-1372-2) contains supplementary material, which is available to authorized users.

## Background

Successive generations of RNA-sequencing technologies have bolstered the notion that organisms produce a highly diverse and adaptable set of RNA molecules. Modern transcript catalogs, such as GENCODE [1], now include hundreds of thousands of transcripts, reflecting pervasive transcription and widespread alternative RNA processing. However, despite years of high-throughput sequencing efforts and bioinformatics analysis, we contend that large amounts of transcriptomic information remain essentially disregarded.

Three major classes of biological events drive transcript diversity. Firstly, transcription initiation occurs at multiple alternative promoters in protein-coding and non-coding genes and at multiple antisense or inter/intragenic loci. Secondly, transcripts are processed by a large variety of mechanisms, including splicing and polyadenylation, editing [2], circularization [3], and cleavage/degradation by various nucleases [4, 5]. Thirdly, an essential, yet often overlooked source of transcript diversity is genomic variation. Polymorphism and structural variations within transcribed regions produce RNAs with single-nucleotide variations (SNVs), tandem duplications or deletions, transposon integrations, unstable microsatellites, or fusion events. These events are major sources of transcript variation that can strongly impact RNA processing, transport, and coding potential.

Current bioinformatics strategies for RNA-seq analysis do not fully account for this vast diversity of transcripts. A widely used approach consists of aligning or pseudo-aligning RNA-seq reads on a reference transcriptome to quantify transcripts [6–8]. Although it may be used in detecting isoform switching events, this analysis is by definition limited to transcripts present in the input reference [9–12]. Another approach attempts to reconstruct full-length transcripts, either reference-based [13] or de novo [14]. Although these protocols can identify novel transcripts, they do not account for true transcriptional diversity as they ignore small-scale variations, such as single-nucleotide polymorphisms, indels, and edited bases, and struggle with repeat-containing transcripts. Yet another class of protocols is devoted to the discovery of specific events, such as splicing events [15–17], alternative polyadenylation events [18], intron retention events [19], fusion transcripts [20, 21], circular RNAs [22], or allele-specific expression [23]. Strategies combining multiple software items for a comprehensive transcriptome analysis [24] are difficult to implement and cannot be truly exhaustive.

Using public human RNA-seq data sets, we show that a large amount of captured RNA variation is not represented in existing transcript catalogs. We propose a new approach to RNA-seq analysis that facilitates the discovery of such events, independently of alignment or transcript assembly. Our approach relies on *k*-mer indexing of sequence files, a technique that recently gained momentum in next-generation sequencing data analysis [7, 8, 25–27]. To identify biologically meaningful transcript variations, our method filters out *k*-mers present in a reference transcriptome and selects those with differential expression (DE) between two experimental conditions; hence its name, DE-kupl. When several *k*-mers represent the same variation, they are merged into a larger contig. As a proof of concept, we applied DE-kupl to RNA-seq data from an epithelial–mesenchymal transition (EMT) model and a variety of human tissues. DE-kupl identified significant numbers of novel events and was able to identify similar events reproducibly in independent RNA-seq experiments.

## Results

### Reference data sets are an incomplete representation of actual transcriptomes

**Fig. 1:**
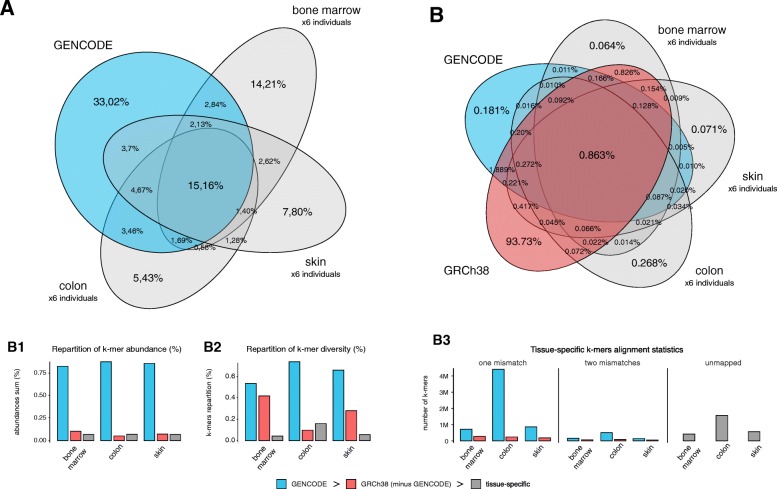
The diversity of 31-mers in RNA-seq libraries exceeds that of reference sequences. **a** Intersection of *k*-mers present in GENCODE transcripts and RNA-seq data from three tissues: bone marrow, skin, and colon. The set of *k*-mers for each tissue was defined as the set of *k*-mers shared by all six individuals. **b** Intersection of *k*-mers present in GENCODE transcripts, the reference human genome (GRCh38), and RNA-seq data (same as in **a**). **b1** Distribution of *k*-mer abundances for each tissue represented in **a** and **b**. *k*-mers shared with GENCODE are labeled as GENCODE. Among other *k*-mers, those shared with the human genome are labeled as GRCh38. The remaining *k*-mers are labeled as tissue-specific. The same procedure was applied in **b2** and **b3**. **b2** Repartition of *k*-mer diversity for each tissue. **b3** Mapping statistics of *k*-mers labeled as tissue-specific in **b2**. These *k*-mers were first mapped to GENCODE transcripts, and unmapped *k*-mers were then mapped to the GRCh38 reference using Bowtie1, with a tolerance of up to two mismatches in a 31-mer

We first analyzed *k*-mer diversity in different human references and high-throughput experimental sequences. Thus, we extracted all 31-nt *k*-mers from sequence files using the Jellyfish program [28]. Figure 1a, b compares *k*-mers from GENCODE transcripts and the human genome reference, with RNA-seq libraries from 18 different individuals [29] corresponding to three primary tissues (six libraries/tissue). To minimize the risk of including *k*-mers containing sequencing errors, for each tissue we retained only the set of *k*-mers appearing in at least six individuals.

Measures of *k*-mer abundance show that *k*-mers are overwhelmingly associated with GENCODE transcripts (Fig. 1 b1). However, when considering *k*-mer diversity, a large proportion of *k*-mers are tissue-specific and not found in the GENCODE reference (Fig. 1a). These tissue-specific *k*-mers may result from sequencing errors, genetic variation in individuals, or novel or non-reference transcripts. The majority of RNA-seq *k*-mers that do not occur in GENCODE are found in the human genome reference (Fig. 1b, b2). This suggests that polymorphisms and errors represent a small fraction of tissue-specific *k*-mers and that many *k*-mers result from expressed genome regions that are not represented in GENCODE. Further scrutiny of tissue-specific *k*-mers shows that many can be mapped to the transcriptome with one substitution. However, for each tissue, there is an average of 1 million *k*-mers that cannot be mapped to either reference (Fig. 1 b3).

**Fig. 2:**
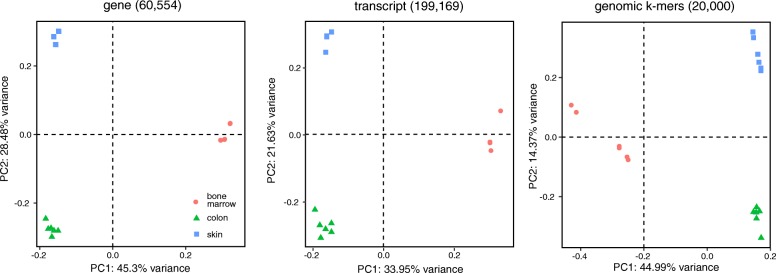
Principal component analysis for non-reference *k*-mers discriminates tissues. Samples are labeled according to their tissues (bone marrow, colon, and skin). PCs were produced with normalized log-transformed counts. For genes and transcripts, counts were generated with Kallisto based on GENCODE V25. Genomic *k*-mers correspond to 20k random *k*-mers from the RNA-seq libraries that did not map to GENCODE transcripts but successfully mapped to GRCh38. PC principal component

Non-reference *k*-mers classify samples as accurately as reference transcripts. We performed a principal component analysis (PCA) of the human tissue samples described above using conventional transcript counts and *k*-mer counts. PCA based on 20,000 randomly selected unmapped *k*-mers was able to differentiate tissues as accurately as PCA based on estimated gene expression or transcript expression (Fig. 2). This illustrates the biological relevance of non-reference transcriptome information that is not accounted for in standard analyses.

**Fig. 3:**
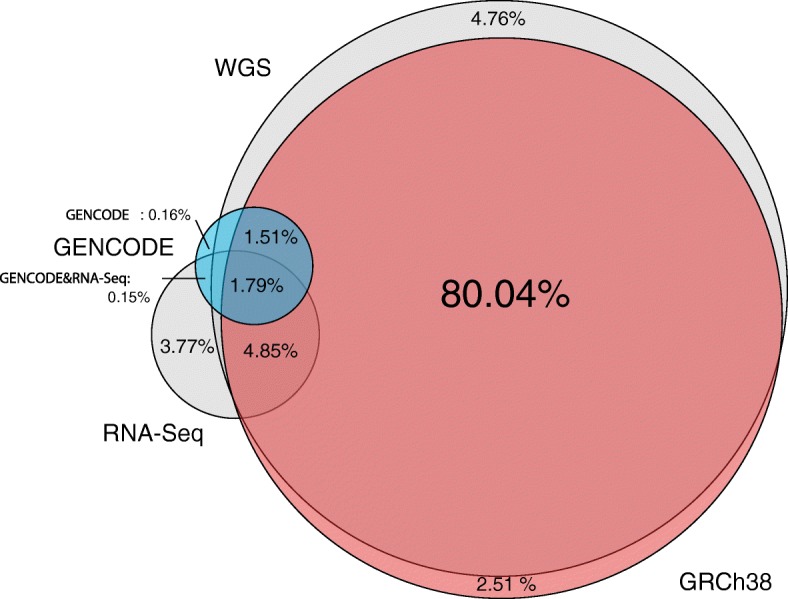
The diversity of non-reference *k*-mers is greater for RNA-seq than for WGS. Intersection of *k*-mers between GENCODE transcripts, the human genome (GRCh38), RNA-seq, and WGS data. RNA-seq and WGS data originate from the same lymphoblastoid cell line (HCC1395). WGS whole-genome sequencing

When comparing RNA-seq and whole-genome sequence (WGS) data from the same individual [30], library-specific *k*-mers are observed much more frequently in RNA-seq than in WGS *k*-mers (Fig. 3). This shows that non-reference sequence diversity is larger in RNA-seq than in WGS. Altogether, these results suggest the existence of a significant amount of untapped biological information in RNA-seq data.

Non-reference *k*-mers may result from the three aforementioned classes of biological events. Specifically, we expect that genetic polymorphism, intergenic expression (e.g., long intergenic non-coding RNA or lincRNA, antisense RNA, expressed repeats, or endogenous viral sequences) and alternative RNA processing (polyadenylation, splicing, and intron retention) are the predominant sources of non-reference *k*-mers. In combination, these genetic, transcriptional, and post-transcriptional events may have a profound impact on transcript function.

### A new *k*-mer based protocol for deriving transcriptome variation from RNA-seq data

We designed the DE-kupl computational protocol with the aim of capturing all *k*-mer variation in an input set of RNA-seq libraries. This protocol has four main components (Fig. 4):

1. Indexing: index and count all *k*-mers (*k*=31) in the input libraries

**Fig. 4:**
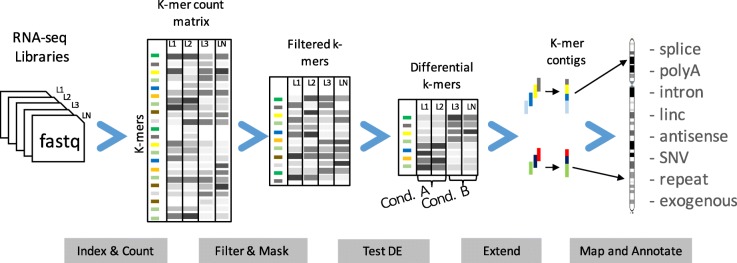
The DE-kupl pipeline for the discovery and analysis of differentially expressed *k*-mers. First, Jellyfish is applied to count *k*-mers in all libraries. *k*-mers counts are then joined into a count matrix and filtered for low recurrence and matching to the reference transcriptome. Normalization factors are computed from raw *k*-mer counts and the differential expression procedure is applied. Finally, overlapping differentially expressed *k*-mers are extended into contigs and annotated based on their alignment to the reference and overlap with annotated genes
2. Filtering and masking: delete *k*-mers representing potential sequencing errors or perfectly matching reference transcripts
3. Differential expression (DE): select *k*-mers with significantly different abundances across conditions
4. Extending and annotating: build *k*-mer contigs and annotate contigs based on sequence alignment.

DE-kupl departs radically from existing RNA-seq analysis procedures in that it performs neither map-first (like Tuxedo suite [31]) nor assemble-first (like Trinity [32]) but instead directly analyzes the contents of the raw FASTQ files, displacing mapping to the final stage of the procedure. In this way, DE-kupl guarantees that no variation in the input sequence (even at the level of a single nucleotide) is lost at the initial stage of the analysis. Even unmappable *k*-mers from repeats, low complexity regions, or exogenous organisms are retained till the final stage and can, thus, be analyzed.

The DE-kupl protocol is detailed in “Methods”. We highlight here some of its key features. First, DE-kupl must accommodate the large size of the *k*-mer index. A single human RNA-seq library contains of the order of 10^7^ to 10^8^ distinct *k*-mers. We selected the Jellyfish tool for counting *k*-mers [28] as it has very fast computing times and allows the storage of the full index on disk for further querying.

A central process in DE-kupl is *k*-mer filtering and masking. Filtering out unique or rare *k*-mers is relatively straightforward and considerably reduces *k*-mer diversity and the number of sequence errors. Masking entails the removal of *k*-mers matching a reference transcript collection. The rationale for this is that the bulk of *k*-mers in RNA-seq data comes from known exons, a form of canonical exon expression ignored in this study as it can be captured efficiently by conventional reference-based protocols [7, 8]. Discarding these *k*-mers enables us to ignore the strong signal caused by known transcripts, allowing us to focus better on expressed regions harboring differences from the reference transcriptome. Depending on the application, masking can be performed using a full annotation such as GENCODE or a simpler transcriptome limited to major transcripts, or skipped altogether.

Two modes are available for the differential analysis of *k*-mers (Additional file 1: Figure S1 and “Methods”). The *t*-test mode is fast and has low sensitivity, i.e., it retrieves only the most significantly DE *k*-mers. The DESeq2-based mode [33] is slower, more sensitive, and is, therefore, recommended for small samples (fewer than six vs six samples). Finally, a *k*-mer extension procedure merges overlapping *k*-mers into contigs and stops as soon as a fork is encountered (i.e., when a contig extremity is overlapped by two different *k*-mers). Rather than producing full-length transcripts, this procedure is intended to group *k*-mers overlapping a single event. Whenever possible, the key steps of the procedure (*k*-mer table merging, *t*-test, and *k*-mer extension) were written in C, enabling the whole procedure to run on a relatively standard computer in a reasonable amount of time.

### Discovery of differential RNA contigs with DE-kupl

**Fig. 5:**
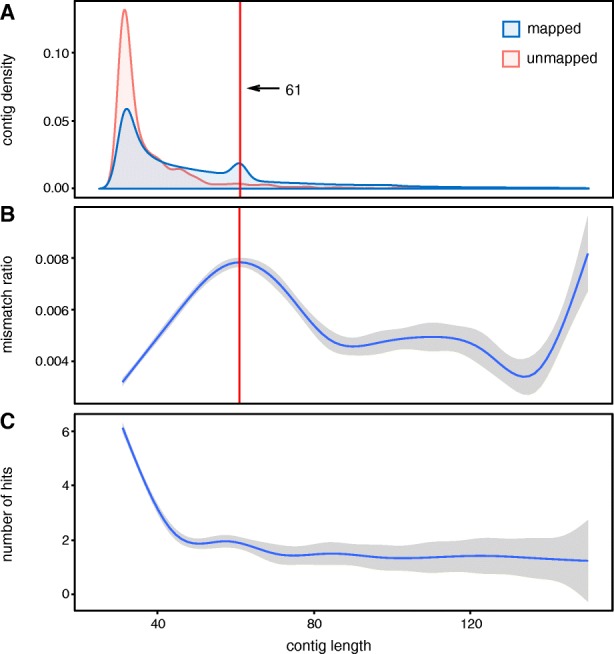
Specificity of differentially expressed contigs. **a** Density plot of contig lengths for mapped and unmapped contigs. The red line indicates contigs built from *k* *k*-mers and likely corresponding to SNVs. **b** Mismatch ratio (number of mismatches/contig size) as a function of contig length. **c** Number of hits in the reference genome as a function of contig length. The **b** and **c** curves were obtained using a smoothing function. SNV single-nucleotide variation

To assess DE-kupl’s capacity to discover novel differential events, we applied it to 12 RNA-seq samples from an EMT cell-line model [34], in which non-small cell lung cancer (NSCLC) cells were induced by ZEB1 expression over a 7-day time course. We compared six RNA-seq libraries from the epithelial stage of the time course (uninduced and day 1) with six libraries from the mesenchymal stage (days 6 and 7). The full DE-kupl procedure was completed in about 4 h in *t*-test mode (single threaded) and 6.5 h in DESeq2 mode (multi-threaded), using eight computing cores, 54 GB RAM, and 7 to 42 GB of hard disk space (Table 1). Recurrence filters efficiently reduced *k*-mer counts from 707 to 92.5M and GENCODE masking further reduced counts to 40.3M. Differential analysis in *t*-test mode eventually retained 3.8M *k*-mers that were assembled into 133,690 contigs (Table 2). The resulting contigs ranged in size from 31 bp (corresponding to an orphaned unextended *k*-mer) to 3.6 kbp, with a major peak of short 31–40 bp contigs and a minor peak around 61 bp contigs (Fig. 5a).

**Table 1:** DE-kupl parameters and resources used for analyzing epithelial–mesenchymal transition data (12 libraries) using the *t*-test or DESeq2 method (GENCODE masking)

Parameter/resources	Value		
nb_threads	8		
min_recurrence	6		
min_recurrence_abundance	5		
pvalue_threshold	0.05		
lib_type	Stranded		
	*t*-test	DESeq2	
Maximum memory usage	54 GB	53 GB	
Maximum disk used (1)	7 GB	42 GB	
Running time (1)	4 h 2 m	6 h 33 m	

(1) excluding reference genome and transcriptome indexing for the annotation step

**Table 2:** DE-kupl pipeline results for the epithelial–mesenchymal transition experiment

Files	Description	Number of *k*-mers		Sizes		
		or contigs				
raw_counts (no filter)	Matrix of *k*-mers counts	707,067,278		(not generated)		
	from all libraries					
filtered_counts.tsv.gz	Matrix of all *k*-mer counts from	92,525,450		1.9 GB		
	all libraries with recurrence filters					
masked-counts.tsv.gz	Matrix of counts after	40,398,848		728 MB		
	GENCODE masking					
		*t*-test	DESeq2	*t*-test	DESeq2	
diff-counts.tsv.gz	Counts with differential	3,813,418	6,102,447	186 MB	510 MB	
	expression test, filtered on					
	adujsted *P* value					
merged-diff-counts.tsv.gz	Differentially expressed *k*-mers	133,690	169,613	3.0 MB	18 MB	
	assembled into contigs					

This is a description of output files sequentially generated by DE-kupl. The numbers of *k*-mers and contigs correspond to the number of lines in each file

Almost all (99.2 %) of the 133k DE contigs mapped to the human genome. Mapping revealed that most 61 bp contigs result from the assembly of 31 overlapping *k*-mers harboring a SNV at every position of the *k*-mer. This phenomenon also causes a higher mismatch ratio for contigs around 61 bp (Fig. 5b). Contigs that do not map to the human genome are generally shorter than mapped contigs (Fig. 5a), indicating a lower signal-to-noise ratio in unmapped contigs. As expected, shorter mapped contigs tend to map at multiple loci more often than longer ones (Fig. 5c). However, 80 % of all contigs are uniquely mapped (not shown).

**Fig. 6:**
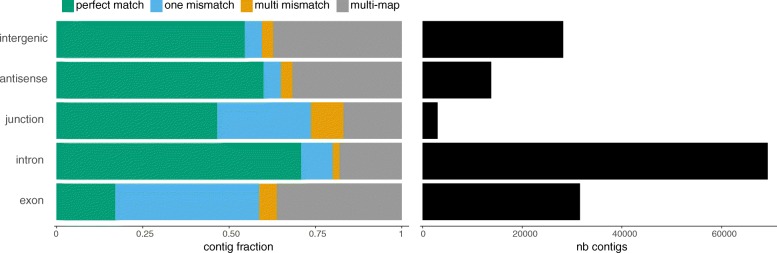
Genomic location of differentially expressed contigs. Contigs are separated by genomic location, according to their overlap with exons, exon–exon junctions, introns, antisense regions of annotated genes, or intergenic regions. Right: Total number of contigs in each class. Left: Contig distribution according to their alignment status. Contigs with a single mapping location are labeled as a perfect match, one mismatch, or multi mismatches. Contigs with multiple mapping locations are labeled as multi-map. nb number of

Analysis of contig locations reveals distinct contig classes. Most contigs are in annotated introns and exons (Fig. 6). However, intronic contigs are predominantly exact matches while exonic contigs are predominantly mismatched. This is due to reference transcript masking: contigs with exact matches to introns are usually not masked, as they do not pertain to a reference transcript, while contigs that match exons are filtered out unless they differ from the reference. This difference might be in the form of SNVs, or through exons extending into flanking intergenic or intronic regions. By the same rationale, contigs mapping to intergenic and antisense regions are depleted in SNVs (Fig. 6), consistent with their location in unannotated lncRNAs and antisense RNAs, while contigs overlapping exon–exon junctions behave like exonic contigs (with a high rate of SNV). However, a significant fraction of exon junction contigs are exact matches, indicating they may correspond to novel junctions.

### Assigning contigs to biological events

We assigned DE contigs generated from the EMT data set to 11 classes of potential biological events, using the rule set described in Table 3. Since intragenic DE contigs may result from a mere over- or under-expression of their host gene and do not necessarily reflect a differential usage (DU) of transcript isoforms, we implemented a simple strategy to distinguish between the two situations based on the expression level of the host gene (see “Methods”). We made this distinction for splicing, polyadenylation, SNVs, and intron retention (Table 3).

**Table 3:** Assignment rules for differentially expressed contigs

	Conditions													
Event class	DU *P* value	Number of junctions	Maps gene	Maps antisense gene	Clipped 3’	Is mapped	SNV	Exonic	Intronic	Number of hits	Contig length	Other rules	Contigs	Loci
Splicing		>0	T			T	F			1			1879	1280
Splicing DU	<0.01	>0	T			T	F			1			391	345
PolyA					≥5	T				1		1	105	95
PolyA DU	<0.01		T		≥5	T				1		1	9	8
lincRNA			F	F		T				1	>200		1061	329
asRNA			F	T		T				1	>200		479	180
SNV DU	<0.01		T	F		T	T	T		1		2	929	680
Intron			T	F	0	T			T	1		3	49897	6689
Intron DU	<0.01		T	F	0	T			T	1		3	10688	3128
Repeats						T				≥5	>50	4	1136	612
Unmapped						F					>50		112	

Each class of event is defined by a set of rules applied to annotated contigs. Other rules refers to the following: (1) contig ends with AAAAA, (2) mean counts >20 in at least one condition and mapped region <10 kb, (3) mapped region <10 kb, and (4) the mapped gene is not differentially expressed. Contigs indicates the number of contigs of each class found in the epithelial–mesenchymal transition experiment. Loci is the number of loci implicated by these contigs (see “Methods”)

*asRNA* antisense RNA, *DU* differential usage, *F* false, *lincRNA* long intergenic non-coding RNA, *PolyA* polyadenylated, *SNV* single-nucleotide variation, *T* true

From the total set of 133k DE contigs (Additional file 1), we extracted about 76,000 contigs matching our rule set for either event class (Table 3). Note that certain events generate multiple contigs. We, thus, further grouped contigs into loci (defined as independent annotated genes or intergenic regions harboring one or more contigs) (Table 3). We describe below the main classes of events identified.

#### Differential splicing

**Fig. 7:**
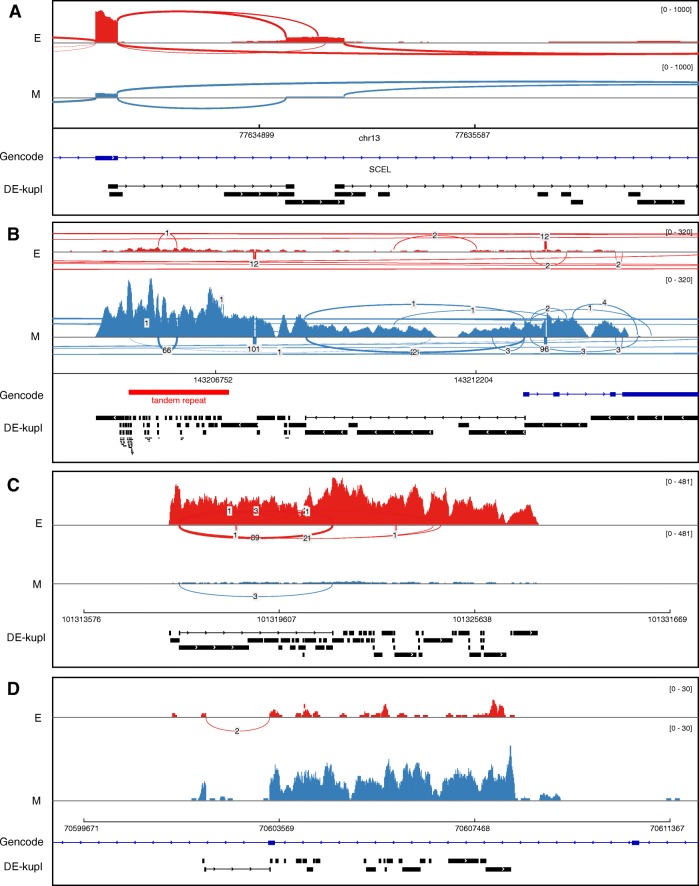
Examples of differentially expressed contigs. Sashimi plots generated from Integrative Genomic Viewer (IGV) using read alignments produced with STAR [52]. Sample SRR2966453 from condition D0 is labeled with E (epithelial). Sample SRR2966474 from condition D7 is labeled with M (mesenchymal). Annotations from GENCODE and DE-kupl differentially expressed contigs are shown at the bottom of each frame. **a** New splicing variant involving an unannotated exon, overexpressed in condition E. **b** Tandem repeat at chr8:143,204-870-143,206,916 (red region) that is overexpressed in condition M vs E. Note that the overexpressed tandem repeat is part of a larger overexpressed unannotated locus. **c** A novel long intergenic non-coding RNA overexpressed in condition E. **d** A novel antisense RNA. RNA-seq reads are aligned in the forward orientation while the gene at this locus is in the reverse orientation. The annotated gene is not expressed. E epithelial, M mesenchymal

An analysis of split-mapped contigs found evidence of potentially novel differential splice variants in 1879 contigs (Table 3, Fig. 7a–c). Furthermore, 391 of these contigs were classified as DU, suggesting that differential splicing at these sites may not be a consequence of DE of the whole gene. Surprisingly, these novel events include a number of subtle variations at 5^′^ and 3^′^ splice sites with 3–15 bp difference from the annotated reference, which escaped prior annotation (see, e,g., Additional file 1: Figure S2).

#### Differential polyadenylation

We extracted all contigs aligned with five or more clipped (e.g., non-reference) bases at their 3^′^ end, and containing five or more trailing A’s. Out of 140 such polyA-terminated contigs, 105 (75 %) contained an AATAAA or variant polyadenylation signal (Additional file 1: Table S1), indicating they result from actual polyadenylated transcripts (Table 3). Note these are not necessarily novel polyadenylation sites since polyadenylated transcripts always create *k*-mers that differ from the reference transcriptome and are, hence, retained by DE-kupl. Indeed, only six of the 105 polyA contigs mapped to intergenic regions. Furthermore, nine polyA contigs were classified as differentially used between the two conditions (Table 3 and Additional file 1: Table S1). Altogether this analysis demonstrates that DE-kupl can capture bona fide polyadenylated transcripts present in the sequencing reads and polyadenylation sites with possible DU.

#### LincRNA

We identified a subset of 1061 DE contigs (329 loci) corresponding to potential lincRNAs (Table 3). The criteria for lincRNAs were contigs of size >200 nt mapped to an intergenic locus. Visual inspection revealed clear lincRNA-like patterns, with contigs clustered into well-defined transcription units with abundant read coverage and evidence of splicing (Fig. 7c, Additional file 1: Figure S3). DE-kupl is, thus, an effective tool for the identification of novel DE lincRNAs.

#### Antisense RNAs

When DE-kupl is applied to stranded RNA-seq libraries (as with the EMT libraries used in this study), the resulting contigs are strand-specific and can, thus, be used for identifying antisense RNAs and for disambiguating loci with intricated expression on both strands. We identified 479 contigs from 180 loci mapping to the reverse strand of an annotated gene (Table 3). These antisense RNAs include very strong cases of DE (Fig. 7d), sometimes combined with apparent repression of the sense gene (Additional file 1: Figure S4).

#### Allele-specific expression

As DE-kupl quantifies every SNV-containing *k*-mer, we set out to exploit this capacity to identify potential allele-specific expression events. We extracted all contigs including an SNV (either a base substitution or indel) and for which DU was predicted (Table 3). This procedure was less than ideal, as we did not explicitly test for a switch in allelic balance between the two conditions. Yet, among the 929 contigs identified, some appeared to display strong apparent changes in allelic balance between the E and M conditions (e.g., Additional file 1: Figure S5). The ability of DE-kupl to capture differential SNV between data sets may be particularly relevant when looking for recurrent mutations in subpopulations.

#### Intron retention and other intronic events

As highly expressed transcripts often carry intronic by-products, we expected DE-kupl to identify many parasitic intronic contigs. Indeed, 49,897 contigs mapped to intronic loci (Table 3). We, thus, focused on intronic *k*-mers for which DU was predicted, indicating intron retention events. This filter identified 10,688 intronic contigs from 3128 different genes. Inspection of the read mapping at these loci revealed clear instances of novel skipped or extended exons (Additional file 1: Figure S6), as well as cases where a specific short intronic region was DE, reminiscent of the pattern observed for intronic processed microRNAs and small nucleolar RNAs [35] (Additional file 1: Figure S7). DE-kupl can, therefore, be used for screening a wide variety of exon and intron processing events in addition to alternative splicing.

#### Expressed repeats

Assessing the expression of human repeats by conventional RNA-seq analysis protocols is difficult, as ambiguous alignments render repeat regions unmappable [36]. Since DE-kupl first measures expression independently of mapping, we were able to collect and analyze differential contigs with multiple genome hits. We found that 7521 contigs larger than 50 nt have multiple hits (data not shown), and 1136 are repeated more than 5 times (Table 3). RepeatMasker [37] found 693 out of these 1136 sequences to match known repeats, mostly long interspersed nuclear elements, long terminal repeats, and short interspersed nuclear elements (Additional file 1: Figure S8). Further inspection showed that most of the remaining multiple-hit contigs correspond to unannotated repeats or low-complexity regions. One of the most striking differential repeats is an unannotated 22×66 bp tandem repeat, located about 2 Mbp from the chromosome 8 telomere. This repeat is found about 50-fold overexpressed in the mesenchymal condition (Fig. 7b, Additional file 1: Figure S9). These results indicate DE-kupl can serve as a screen for DE or activation of endogenous viral sequences and other repeat-containing transcripts.

#### Unmapped contigs

Finally, we analyzed DE contigs that did not map to the human genome. Unmapped contigs may result from transcripts produced by rearranged genes or by exogenous viral genomes and could, thus, be highly relevant biologically. In principle, DE-kupl is able to detect such events when levels of RNA vary across samples. In this test set, where all samples come from an in vitro cell line, we did not expect to observe this phenomenon. Indeed, out of 112 unmapped contigs of size >50 bp (Table 3), the vast majority (76 %) correspond to vector sequences overexpressed in the M condition (data not shown), indicating that these contigs come from the expression vector used for EMT induction. The remaining unmapped contigs correspond to a GA tandem repeat and several non-human primate sequences.

### Impact of transcriptome masking

Using GENCODE as a reference transcriptome removed about half of the *k*-mers (Table 2). We analyzed the impact of using different reference transcriptomes on differential *k*-mer and contig calls. We ran DE-kupl on the EMT data set using a lightweight masking transcriptome limited to major transcripts (1 transcript/gene, see “Methods”) and in the absence of masking (Additional file 1: Table S2). Masking with the lightweight transcriptome had a moderate impact on the number of DE *k*-mers and contigs (1.6- and 1.4-fold increase, respectively). However, a complete bypass of the masking procedure caused a large increase in DE *k*-mers and contigs (3.4- and 2.4-fold, respectively). Importantly, less stringent masking produced longer contigs (Additional file 1: Figure S10) and a higher number of detected events, especially in the splicing and intron categories (Additional file 1: Table S3). These results indicate that, in a typical DE-kupl use case, lightweight masking may be the preferred option, returning a higher number of events for little additional computational cost.

### Comparison with specialized tools

We compared DE-kupl events with predictions from two specialized tools. Since DE-kupl reports only events with DE, the protocols compared should involve an event-calling stage combined with a differential filter. IRFinder [19] and KisSplice [15] predict intron retention and de novo differential splicing events, respectively. Both pieces of software report changes in relative inclusion, i.e., variants whose proportions vary between conditions. Therefore, their results can be compared with differential (DU) introns and splice sites from DE-kupl. After running IRFinder on the EMT data set, we observed a strong enrichment in IRFinder predictions among the top DE-kupl intron retention events (Additional file 1: Figure S11). Conversely, 68 % of IRFinder intron retention events were predicted by DE-kupl as intron DU (DU *p* value <0.05) and this fraction rose to 80 % among the 100 top ranking IRfinder predictions (Additional file 1: Table S4). A comparison with KisSplice showed a similar enrichment in KisSplice predictions among the top DE-kupl splice events (Additional file 1: Figure S12). While only 36.4 % of all KisSplice predictions were present among the total DE-kupl splice events, DE-kupl predicted as splice DU (splice events with DU) 82 of the top 100 KisSplice predictions (Additional file 1: Table S4). These results suggest DE-kupl is able to recall the majority of top ranking predictions made by two specialized tools.

### DE-kupl event detection reproduced across independent data sets

**Fig. 8:**
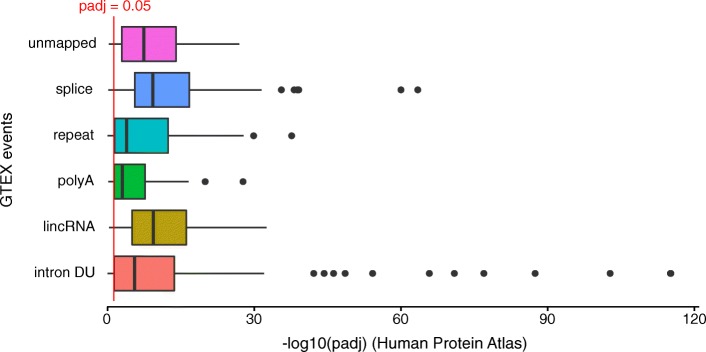
Validation of DE-kupl events across independent data sets. Altogether, 550 differentially expressed contigs from six different event classes (intron with differential usage, lincRNA, polyA site, repeat, splice site, and unmapped) were identified using DE-kupl on GTEx libraries from two human tissues (skin and colon). A representative *k*-mer from each contig was then tested for differential expression in the skin and colon libraries in the Human Protein Atlas. Box plots represent distributions of DESeq2 adjusted *p* values for all *k*-mers in the different classes. The red line shows the adjusted *p* value cutoff of 0.05. DU differential usage, lincRNA long intergenic non-coding RNA, padj adjusted *p* value, polyA polyadenylation

We sought independent validation of DE-kupl findings with two distinct human RNA-seq data sets, from the Genotype-Tissue Expression (GTEx) [38] and the Human Protein Atlas (HPA) [29]. DE contigs were first obtained by running DE-kupl on eight colon vs eight skin libraries from GTEx. Events were classified as above into intron retentions, lincRNAs, polyadenylation sites, repeats, splice sites, and unmapped. The 100 top events from each class (50 for class unmapped) were extracted and their *k*-mer labels saved as a sequence file. We then counted the occurrence of each *k*-mer in the colon and skin libraries from the HPA project and applied DEseq2 [33] to evaluate the significance of the expression change between colon and skin (see “Methods”). Altogether, 79 % of the 550 DE *k*-mers identified by GTEx were also significantly DE in the HPA data (Fig. 8). Each event class showed clear reproducibility, with particularly strong effects for lincRNAs and splice variants. This demonstrates that novel events identified by DE-kupl are reproducible across independent data sets despite independent RNA extraction, library preparation, and sequencing protocols.

## Discussion

In contrast to popular RNA-seq analysis software, DE-kupl does not attempt full-length transcript assignment or assembly but focuses on local transcript variations instead. Indeed, we do not consider full-length transcript analysis to be realistic when screening for unspecified RNA variation, since the combinatorial nature of genomic, transcriptomic, and post-transcriptomic events would require an indefinitely expanding transcript catalog. In this sense, DE-kupl is closer in spirit to methods analyzing local RNA-seq coverage such as RNAprof [39] and DERfinder [40], with the notable difference that DE-kupl does not involve mapping and, thus, avoids mapping-related pitfalls while considerably widening the range of detectable events. Another important benefit of the *k*-mer strategy is that *k*-mers representing events of interest can be used efficiently to assess the occurrence of similar events in the huge public compendium of RNA-seq data.

In this proof-of-concept study, we analyzed RNA-seq libraries from a small number of individuals and from a single cell line. We expect *k*-mer diversity to rise significantly with the number of individuals included in the analysis. However, preliminary tests with over 100 libraries from The Cancer Genome Atlas [41] show a sublinear growth in the number of *k*-mers with the number of libraries (Additional file 1: Figure S13), which suggests there is good scalability of the DE-kupl concept. Analysis of large-scale patient RNA-seq data opens exciting perspectives. For instance, the ability of DE-kupl to detect genetic variation and RNA expression/processing events simultaneously may serve as a basis for studying genotype/phenotype relations. Analysis of patient RNA-seq data may also reveal event classes not studied in this work, such as fusion transcripts and circular RNAs.

## Conclusion

*k*-mer decomposition followed by filtering, masking, and DE analysis is a novel way of analyzing RNA-seq data. It can detect a wider spectrum of transcript variation than previous protocols. DE-kupl explores all *k*-mers in the input RNA-seq files (vs only *k*-mers from annotated transcripts in recent software [7, 8]), which potentially requires substantial computational time and memory resources. Using the Jellyfish *k*-mer indexing software and C-programming code for the key table manipulation, we achieved time/memory requirements on par with popular mapping-based software for similarly sized data sets. A key aspect of our protocol that rendered a full *k*-mer analysis tractable was the application of successive filters for rare *k*-mers, reference transcripts, and DE, which altogether resulted in a 200-fold reduction in *k*-mer counts. These filters are not only useful for technical considerations (they reduce run times and enable us to get rid of most sequence errors), but also they allow the user to focus on *k*-mers that (i) vary significantly between the conditions under study and (ii) encompass events that would not be captured by conventional reference-based protocols.

We showed that DE-kupl is able to detect a wide range of differential transcription and RNA processing events. Although specialized software may perform better at assessing specific event classes, such as differential splicing, no method known to the authors provides such a comprehensive screen. As differential RNA-seq analysis is often conducted with an exploratory spirit, we argue that it is preferable to cast a wide net with no preconceptions for target events, using DE-kupl along with a conventional gene-by-gene DE analysis. Note that DE-kupl might also be an interesting option for exploring other types of next-generation sequencing data, such as small RNA-seq, ChIP-seq, or whole-exome/genome sequencing, after adjusting its parameters and event annotation rules.

## Methods

### Characterization of *k*-mer diversity in human RNA-seq libraries

RNA-seq data for bone marrow, skin, and colon from 18 individuals (six replicates per tissue) were retrieved from the HPA project [29] (E-MTAB-2836). We counted *k*-mers in each RNA-seq and reference sequence set using Jellyfish (2.2.6), with options *k*=32 and -C (canonical *k*-mers). The *k*-mer list for each tissue (Fig. 1a, b) was produced by merging counts for all six samples and conserving only those found in all replicates.

For mapping statistics (Fig. 1 b3), we extracted *k*-mers specific to each tissue and mapped them to the Ensembl 86 transcript reference using Bowtie (version 1.1.2). Unmapped *k*-mers were mapped a second time with Bowtie to the GRCh38 genome reference. Reads with three or more mismatches are not mapped by Bowtie and, therefore, are considered as unmapped.

The intersection of *k*-mers between RNA-seq and WGS data (Fig. 3) is based on the transcriptome and genome of lymphoblastoid cell lines [30]. *k*-mers were counted in these libraries with the same procedure as above. To reduce noise from sequencing errors, *k*-mers with only one occurrence were filtered out.

### DE-kupl implementation

The DE-kupl pipeline (Additional file 1: Figure S14) is implemented using the Snakemake [42] workflow manager (v3.10.1). There is a configuration file containing the location of FASTQ files, the condition of each sample, as well as global parameters such as *k*-mer length, CPU number, maximum memory, and other parameters for each step of the pipeline, as described hereinafter.

#### *k*-mer counting

Raw sequences (FASTQ files) are first processed with the jellyfish count command of the Jellyfish software, which produces one index (a disk representation of the Jellyfish hash table) for each sequence library. For stranded RNA-seq libraries, reads in the reverse direction relative to the transcript are reverse-complemented, ensuring the proper orientation of *k*-mers. At this point, for each library, only *k*-mers having at least two occurrences are recorded (a user-defined parameter). Once a Jellyfish index is built, we use the jellyfish dump command to output the raw counts in a two-column text file, which contains at each line a *k*-mer and its frequency of occurrence. Raw counts are then sorted alphabetically by *k*-mer sequence with the Unix sort command.

#### *k*-mer filtering and masking

All sample counts are joined together using the dekupl-joinCounts binary to produce a single matrix with all *k*-mers and their abundance in all samples. Given an integer *a*≥0, we define the *recurrence* of a *k*-mer *x* as the number of samples where *x* appears more than *a* times, i.e.,

$$\text{recurrence}(x,a) = \sum_{i=1}^{n} \mathbbm{1}_{\{x_{i} > a \}}, $$

where *n* is the total number of samples and *x*_*i*_ is the number of times the *k*-mer *x* appears in sample *i*. The *k*-mer filtering step involves two user-defined parameters (an integer min_recurrence_abundance and an integer min_recurrence), such that a *k*-mer *x* is filtered out if

$${{} \begin{aligned} &\text{recurrence}(x,\texttt{min\_recurrence\_abundance})\\\qquad& < \texttt{min\_recurrence}, \end{aligned}} $$

i.e., if the *k*-mer *x* appears more than min_recurrence_abundance times in fewer than min_recurrence of the samples. Usually min_recurrence is set to the number of replicates in each condition, and min_recurrence_abundance is set to 5.

The masking process uses the same Jellyfish-based procedure to create the set of *k*-mers appearing in the reference transcriptome and to subtract this set from the experimental *k*-mers. Masking can be performed using any reference transcriptome. Here, we use either GENCODE V.24 or a simplified transcriptome containing one major transcript per gene, built as follows. Principal transcripts for protein-coding genes are extracted from the APPRIS database [43]. When several isoforms have the same principal level, the longest one is selected. All non-coding RNA transcripts are extracted from GENCODE and the longest transcript is retained when isoforms are present. The lightweight transcriptome, referred to as 1 transcript/gene, is produced by merging the protein-coding and non-coding RNA transcript sets.

#### Differential *k*-mer expression

Prior to differential analysis, we compute normalization factors (NFs) using the median ratio method [44] with the table of *k*-mers after the recurrence filter. For each sample, the NF is the median of the ratios between sample counts and counts of a pseudo-reference obtained by taking the geometric mean of each *k*-mer across all samples. To avoid dealing with the complete table of *k*-mers, we extracted a random subset of 30 *%* of the *k*-mers and computed NFs for this subset. Computing NFs for the complete table of *k*-mers, for the table of *k*-mers after the recurrence filters and reference masking, or for the table of transcript abundances produced by Kallisto v0.43.0 [7] resulted in similar values (Additional file 1: Figure S15).

Two options are implemented for the differential analysis (Additional file 1: Figure S1). The first option is to apply a *t*-test for each *k*-mer on the log-transformed counts, normalized with the previously computed NF. Transformation of raw counts in conjunction with linear model analysis has been successfully used for differential analysis of counts [45]. We perform the *t*-test independently on each *k*-mer and avoid complex variance modeling strategies to reduce the execution time of the analysis. The *t*-test option has been implemented in C in the dekupl-TtestFilter binary. Note that this *t*-test option is not appropriate for small samples [46]. To increase the power of the analysis, in particular for small samples (typically less than six vs six libraries), we strongly advise the use of the second option based on a generalized linear model, implemented in the R package DESeq2 [33]. On top of modeling raw counts (normalization or prior log-transformation of the counts is not required), this approach shares information across *k*-mers to improve variance estimation and the differential analysis results. However, given the large number of *k*-mers, we do not apply this approach to the complete matrix of *k*-mer counts. We divide the matrix of *k*-mer counts into random chunks of approximately equal size (around 1 million *k*-mers) and apply the DESeq2 model independently on each chunk. Previously computed NFs are used as an input to the method for each chunk, and are not computed independently on each chunk. Raw *p* values, unadjusted for multiple testing, are collected as an output for each chunk, and merged into one single vector containing the raw *p* values for all *k*-mers to test. Subsequently, raw *p* values obtained from either the *t*-test or the DESeq2 test are adjusted for multiple comparisons using the Benjamini–Hochberg procedure [47] and *k*-mers with adjusted *p* values above a user-set cutoff are filtered out.

#### *k*-mer extension

DE *k*-mers that potentially overlap the same event (i.e., all *k*-mers overlapping a splice junction or SNV) are joined together using a technique inspired by de novo assembly. The *k*-mer extension procedure, called mergeTags, works as follows. We first identify all exact *k*−1 prefix–suffix overlaps between *k*-mers. We consider only *k*-mers that overlap with exactly one other *k*-mer, and merge all pairs of *k*-mers involved in such overlaps into *contigs*. For example, given a set of *k*-mers {ATG, TGA, TGC, CAT}, the following contigs are produced: {CATG, TGA, TGC}. We repeatedly merge contigs that overlap exactly over *k*−1 bp with exactly one other contig. We then repeat this extension process with *k*−2 exact prefix–suffix overlaps, using as input the contigs produced at the previous step, and so forth for increasing values of *i* such that *k*−*i*>15 bp. The effect of varying *i* on the final number of contigs is presented in Additional file 1: Figure S16. A minimal overlap *k*−*i*=15 was empirically selected. Finally, a set of DE contigs is produced with each contig, being labeled by its constitutive *k*-mer of lowest *p* value. This extension procedure is implemented in C in the dekupl-mergeTags binary.

#### Contig annotation

Finally, DE contigs are annotated to facilitate biological event identification. Contigs are first aligned using BLAST [48] against Illumina adapters. Contigs matching these adapters are discarded. Retained contigs are further mapped to the reference Hg38 human genome using the GSNAP short read aligner [49] (v2017-01-14), which provided the best speed/sensitivity ratio for aligning both short and long contigs in internal tests (data not shown). GSNAP is used with option -N 1 to enable identification of new splice junctions. Contigs not mapped by GSNAP are collected and re-aligned using BLAST.

Alignment characteristics are extracted from GSNAP and BLAST outputs. Alignment coordinates are compared with Ensembl (v86) annotations (in GFF3 format) using BEDTools [50] and a set of locus-related features is extracted. The final set of annotated features (Additional file 1: Table S5) is reported in a contig summary table. The annotation procedure generates two additional files: a per locus summary of contigs (one line per genic or intergenic locus), and a BED file of contig locations that can be used as a display track in genome browsers. In the per locus table, a locus is defined as an annotated gene, the genomic region located on the opposite strand of an annotated gene, or the genomic region separating two annotated genes. The table records the number of contigs overlapping each locus as well as the contig with the lowest false discovery rate for this genomic interval.

Parallel to *k*-mer counting, filtering, and masking, we analyze the RNA-seq data libraries using a conventional DE protocol. Reads are processed with Kallisto [7] to estimate transcript abundances. Transcript-level counts are then collapsed to the gene level and processed with DESeq2 [33] to produce a set of DE genes. This information is stored in the contig summary table and used later to define events with DU (Table 3).

#### DE-kupl run on EMT data

DE-kupl was run using RNA-syeq libraries from reference [34]. The DE-kupl parameters were kmer_length 31, min_recurrence 6, min_recurrence_abundance 5, pvalue_threshold 0.05, lib_type stranded, and diff_method Ttest, with the GENCODE reference. Output files are provided in Additional file 1. The DE-kupl contig summary table was analyzed interactively using R commands to extract lists of contigs based on the filtering rules described in Table 3. Visualization of selected contigs was performed with IGV [51], using the BED file produced by DE-kupl and read mapping files produced by STAR [52].

For comparison with KisSplice and IRFinder, DE-kupl was used with the same parameters as above, except for diff_method DeSeq2 and the 1-transcript/gene reference. KisSplice scripts were run in the following order: kissplice (v2.4.0) > kisstar (v2.5.3a) > kiss2ref (v1.0.0) > kissDE (v1.5.0). The final kissDE step provides the list of splice variant pairs with significant change in percentage inclusion across conditions. IRFinder (v1.2.3) was run with parameters IR ratio > 0.1 and intron coverage > 10. IRfinder outputs a list of introns with differential inclusion levels across conditions. The outputs of both IRfinder and KisSplice were filtered to retain only events matching annotated genes.

#### Validation in independent data sets

DE-kupl was applied to eight skin and eight colon libraries from GTEx [38] using parameters kmer_length 31, min_recurrence 6, min_recurrence_abundance 5, pvalue_threshold 0.05, lib_type unstranded, diff_method Ttest, and reference_transcriptome Gencode. DE-kupl contigs were interactively classified using R commands, applying the same rules as in Table 3. Classes antisense RNA and SNV-DU were excluded since identification of antisense RNA is not possible using the unstranded GTEx and HPA libraries, and we had no reason to expect common SNVs with DU in this data set. DE contigs were sorted by fold-change. The *k*-mer labels of the top 100 DE contigs in each class were extracted (50 for class unmapped due to the fewer events). GTex *k*-mers were then sought in the six skin and six colon libraries from HPA described above [29] (E-MTAB-2836). The *k*-mers were counted in each library using Jellyfish with options *k*=31 and -C (canonical *k*-mers) as GTEx data were unstranded. All *k*-mers selected from the GTEx analysis were queried against the Jellyfish databases using the jellyfish query command. Finally, the extracted *k*-mers counts were processed with DESeq2 [33] and the resulting adjusted *p* values were plotted for each event class (Fig. 8).

## Additional file

Additional file 1 Supplementary **Tables S1–S5**, Supplementary **Figures S1–S16**. (PDF 3112 kb)
