# Supplementary material for: DE-kupl: exhaustive capture of biological variation in RNA-seq data through k-mer decomposition
Source: Genome Biol. 2017 Dec 28;18:243. doi: 10.1186/s13059-017-1372-2 (PMC5747171; doi:10.1186/s13059-017-1372-2)
Supplement: Additional file 1 — Supplementary Tables S1–S5, Supplementary Figures S1–S16. (PDF 3112 kb) [file 13059_2017_1372_MOESM1_ESM.pdf]

Supplemental Materials.
DE-kupl: Exhaustive capture of biological variation in RNA-seq data through k-mer decomposition

Table S 1: Potential differential polyadenylation sites

Contig (clipped, polyA signal in bold)	chromosome	start	end	HUGO_ID_gene	log2FC
TC**AATAAA**CTTTTTTTGTCTGTTGATAATAAAAAAA	chr1	153561340	153561368	S100A2	-3.483503
GACCTGCC**AATAAA**AATTTATGGTCCAAGGAAAAAAA	chr17	41523619	41523647	KRT19	-3.239867
GCCCTTGA**AATAAA**CCTTAGCTGCCCCACAAAAAAA	chr19	35503304	35503332	DMKN	-3.202741 (1)
GAATCTTC**AATAAA**ATGCTTTTGTCATTCAAAAAAAA	chr12	52806549	52806577	KRT4	-3.050761
TCCTCTC**AATAAA**GTTCCCCTGTGACACTCAAAAAAA	chr1	26864428	26864456	SFN	-2.935715
CACAGCCCAGCCCACCCACTGGGT**AATAAA**AGTGGTTTGTGGAAAAAAAA	chr15	40857597	40857655	SPINT1	-2.753893
GACAGGGCTC**ATTAAA**CCTTCCTCTCTGCCAAAAAAA	chr19	38304975	38305003	C19orf33	-2.740276 (1)
TGTATTC**AATAAA**CTTTTTTTGTCTGTTGAAAAAAAA	chr1	153561352	153561373	S100A2	-2.672970
CTTCAAAATG**CATAAA**CCTGTTACAATGTTAAAAAAAA	chr1	46185930	46185958	TSPAN1	-2.604010
TATGTCTCT**AATAAA**GTATTCATTTGATAAAAAAAA	chr8	119245645	119245673	MAL2	-2.322323
TGTTC**AATAAA**GGAGACAAGTGCTTTCCCAAAAAAAA	chr2	85394767	85394795	CAPG	-2.273628
CCACTGA**ATTAAA**GTCTTGGAAGAATTTTAAAAAAAA	chr11	106414229	106414257	none	-2.259111
GCACTC**AATAAA**GAATGAATTTTATTATGAAAAAAAA	chr19	50958631	50958659	KLK6	-2.239635
AAAAGATA**AATAAA**TATTTGTTCATTTCAAAAAAA	chr6	70399389	70399417 R	P11-462G2.1	-2.173980 (1)
TTGTTCAT**AATAAA**AGTGAAGTGAATCTGAAAAAAAA	chr10	100364798	100364826	SCD	-1.999393
AGGGCTC**ATTAAA**CCTTCCTCTCTGCCTTCAAAAAAAA	chr19	38304978	38305006	C19orf33	-1.996551
AACTTATTTGTACATTTTTTTTTC**AATAAA**ACTTTTCCAATGACAAAAAA	chr11	75572732	75572783	SERPINH1	1.976234
G**AATAAA**CTTAGCCATTTCGTACACATGGAAAAA	chr6	52264015	52264042	MCM3	-1.965712
AGTTGAT**AATAAA**CCTTTCTGAGATGCAAAAAAA	chr2	131483974	131484000	MZT2A	-1.916548
GCCTG**AATAAA**GTAATGCCTCCGCTTCAAAAAAAA	chr1	156668763	156668791	NES	1.914198
ATG**ATTAAA**CTTGGGTATTGAGATTAAAAAAAA	chr6	28123007	28123031	ZSCAN16-AS1	1.835452
TTGTTTC**ATTAAA**AAGAAGCACTTTATGTAAAAAAAA	chr17	75135984	75136012	HN1	-1.786158 (1)
CAGGAGCTC**AATAAA**TGTTTGTTGCATGAAAAAAAA	chrX	119663203	119663222	none	-1.761105
AACTCC**AGTAAA**GACACTCTTGTCCTTGGAAAAAAA	chr8	144412414	144412442	SLC39A4	-1.748694
GAA**CATAAA**GAATCTATCTGCTGTTAAAAAA	chr19	14477762	14477785	GIPC1	-1.684051
CTTCTGTTTA**AATAAA**AGTGGCCTGGAAAAAAAA	chr1	54849633	54849659	DHCR24	-1.642438
CAAGA**AATAAA**CTCGTAACCCTGTCCTTCAAAAAAAA	chr1	151364304	151364332	SELENBP1	1.626969
GATGCTGA**AATAAA**CTAGGGTTTTGGCCTGAAAAAA	chr14	103519669	103519697	CKB	-1.624048
TTTCCAT**AATAAA**ATAGAAATGTGTGTAAAAAAA	chr17	81887844	81887870	ALYREF	-1.472288
GTCTGATTT**AATAAA**ATACTTAAACACTGAAAAAAAA	chr3	196049284	196049312	TFRC	-1.454169
TTTTAT**AATAAA**TGTTTATTTTCACATTGAAAAAAAA	chr1	160996194	160996222	F11R	-1.431924
GTGGAC**AATAAA**CAGTATTATCAAAGAGAAAAAAAA	chr17	78852977	78853005	TIMP2	1.429344
AA**AATAAA**AAGCCTCAGTGACCCATGAGAATAAAAAAA	chr1	8861000	8861030	ENO1	-1.408636 (1)
GGATTTGTTTC**ATTAAA**AAGAAGCACTTTAAAAAAAA	chr17	75135988	75136016	HN1	-1.389289 (1)
**ATTAAA**ATTACATTGAGGTTCCTAAAAAAAAAA	chr17	78971255	78971277	LGALS3BP	1.362211
**AATAAA**GTTGCTGTTGACTTTCTTTTTGAAAAAAAA	chr17	82294882	82294910	RP13-516M14.1	1.359061
CAGACATGA**AATAAA**AGCTTTGCAAAGGCAAAAAAAA	chr6	158765748	158765776	EZR	-1.333909
ATACAT**AATAAA**AGACATTGTTGCATGGCTAAAAA	chr5	131159292	131159320	HINT1	-1.322379
TAAGCATTTTTTTTTCCTAA**AATAAA**CTCAAATTTATCCTCAAAAAAAAA	chr14	23645583	23645639	DHRS2	-1.314045
ACTCCAGCCTGGGTGACAGAGCCAGACCCCGTCTTAAA**ATTAAA**AAAAAA	chr11	93758931	93758975	C11orf54	1.300124
CTGCAGAG**AATAAA**AGGACCACGTGCAATAAAAAAA	chr16	70252390	70252418	AARS	-1.273876
CCTGAGA**AATAAA**AAGCCTGTCATTTCAAAAAAAA	chr17	78224671	78224699	BIRC5	-1.273051
TTTATT**AATAAA**AGAATCTTTAACTTTAAAAAAA	chr16	3022355	3022376	TNFRSF12A	-1.272306
TGTT**AATAAA**ATTGATGTTTCCATTTTAAAAAAAA	chr10	109406900	109406924	none	-1.261583
TGTAC**AATAAA**TTTTTTTTGGTCAAATTTAAAAAAAA	chr1	153534599	153534627	S100A6	-1.253663
CCCGGTTCC**AATAAA**GAGACTTGGGCTGAAAAAAA	chr14	24138965	24138992	EMC9	-1.244601
TTGATGTTT**AATAAA**GTAGAAAGCACTGTCAAAAAA	chr2	222571745	222571773	FARSB	-1.236854
GCTTATC**AATAAA**GAATATTGAGCCTGGAAAAAAA	chr11	67351548	67351576	POLD4	-1.235038
TGCTGA**AATAAA**CTAGGGTTTTGGCCTGCCAAAAAAAA	chr14	103519667	103519695	CKB	-1.228564
CATC**AATAAA**TACTTTAACTTCCAAAAAAAAAAA	chr17	22360389	22360413	none	1.217866
AACAAATGG**AATAAA**CTTGAATTGTGCTACAAAAAAA	chr7	135926760	135926789	MTPN	-1.175806 (1)
ACAGAATG**AATAAA**CCTCTTTATATTTGCAAAAAAAA	chr14	104759626	104759654	SIVA1	-1.157612
TTG**AATAAA**TAAAAACTCGTTTGTCTTGTCAAAAAA	chr14	74479947	74479975	NPC2	-1.155310
AGCTTCCT**AATAAA**TCCTTGCCAGACTTAAAAAAA	chr1	23788204	23788232	PITHD1	-1.147161
TGGTGGGTAATTGTAC**AATAAA**TTTTTTTTTGGTCAAATTTAAAAAAAAA	chr1	153534599	153534638	S100A6	-1.143007
TTGAAACAA**ATTAAA**ATATGCTGCATTTGAAAAAAA	chr2	200478502	200478530	SPATS2L	-1.136603
ATAATA**AGTAAA**TAACTTGTCTGTAAAAAAA	chr19	40378460	40378483	PLD3	1.124554
AAAAAAA**GTAAAA**CTTTGCTAATATTAAAAAAAA	chr22	42618813	42618839	CYB5R3	1.118937
AAC**AATAAA**ATTCTGTTAGCCAGAAAAAAAA	chr10	71816298	71816320	PSAP	1.118369
GTTTACAACAA**AATAAA**GTATTCACTACCAAAAAA	chr3	149369022	149369050	TM4SF1	-1.104563
TAGAGA**AATAAA**ATATGGACTTCCAATCTAAAAAA	chr19	53223918	53223943	none	-1.100456
GC**ATTAAA**TCTATTTGCCATTTCTGAAAAAA	chrX	54815991	54816015	MAGED2	1.091271
ATGCCCTGCC**ATTAAA**TCCGCAAACAGCCAAAAAAAA	chrX	154531391	154531418	G6PD	-1.078189
GCACTCCAGCCTGGACAACAAGAGCAAAACTCTGTCTC**AATAAA**AAAAAA	chr3	75770343	75770482	none	1.067491
GCCCCCTC**ATTAAA**ATTCACGTTCCCAAAAAAAAA	chr10	86970706	86970731	ADIRF	-1.032081
CCCATGTGAGGG**AATAAA**CCTGGCACTAGGAAAAAAA	chr12	6870800	6870828	TPI1	-1.026203
CACCTCCTGC**AATAAA**ATAGTAGCATCGGCAAAAAAAAAA	chr20	23633657	23633685	CST3	1.021575
GCACATTTA**AATAAA**ATGTTTCTAACTAGAAAAAAA	chr2	38744893	38744921	SRSF7	-1.016371
CCGCC**AATAAA**CTTCAATGGTTTCTACTGAAAAAAA	chr8	22006557	22006585	XPO7	-0.982720
GAGTT**AGTAAA**CCTCTGTACTACAAAAAAAA	chr14	22771762	22771784	OXA1L	0.969875
TTATGGA**ATTAAA**TTGGGTACAGCTTCAAAAAAAA	chr20	45898792	45898820	CTSA	0.942401
TTTTTTGTTA**ATTAAA**TGTTTTGGTCAGAAAAAAA	chr1	161100561	161100589	PFDN2	-0.940090
TATTG**ATTAAA**AAAGCACTTGCTTGATGTAAAAAA	chr1	33013044	33013072	AK2	-0.928627
GG**AATAAA**GTTTTGCTTCCATTCAAAAAAAA	chrX	154348531	154348552	FLNA	0.911024
G**AATAAA**CCCAGAAAATACAGTTAAAAAAAA	chr16	67934506	67934530	CTC-479C5.12	0.907551
GACTCTG**TATAAA**ATGGTTGGGAAGCTGCAAAAAA	chr19	49015898	49015926	RUVBL2	-0.905895
GGGGGCATG**AATAAA**GGCTACAGGCTCCAAAAAAA	chr1	112701131	112701159	RHOC	-0.881874
GGCCTC**AATAAA**GTTTGTGTTTATGCCACCAAAAA	chr8	144789767	144789795	RPL8	-0.880393
CCAGGACC**AATAAA**ATTTCTAAGAGAGCTAAAAAAAAA	chr11	67586625	67586653	GSTP1	-0.876523
TGTGA**AATAAA**ATTGTTTCACCGCCTGGTAAAAAAAA	chr11	14504879	14504904	RP11-140L24.4	-0.872538
GTATGTC**AATAAA**CAACAGCTGAAGCACCAAAAAAAA	chr15	72199029	72199056	PKM	-0.867397
CTTTTC**ATTAAA**GAATGTTTTGGAACTTTAAAAAAA	chr1	156137838	156137866	LMNA	-0.854544 (1)
GTAATCGTGCA**AATAAA**CGCTCACTCCGAAAAAAA	chr19	2273213	2273241	OAZ1	-0.846481
CCCGTTTTACC**AATAAA**CTGAGCGACCCCAAAAAA	chr20	63488014	63488042	EEF1A2	-0.821529
ATTGT**TATAAA**ATGTAGTACTAAATCTGGAAAAAA	chr22	41674808	41674835	SNU13	-0.813005
GTC**AATAAA**ACTGCCCCAGTTTCCCCTTGAAAAAAAA	chr6	33697568	33697596	UQCC2	-0.804764
CTCAA**AATAAA**AAGCCTCAGTGACCCATGAAAAAAAA	chr1	8861005	8861033	ENO1	-0.774743
TTGTA**AATAAA**GTCTATTTTTCTCCCGTGAAAAAAAA	chr11	72002864	72002892	NUMA1	0.770931
CGACCATCTG**ATTAAA**ATGCTTCCTCCCAAAAAAA	chr17	51162005	51162032	NME1	-0.749396
CATTGT**ATTAAA**AGAATCTGTTTAAACAACAAAAA	chr5	10264986	10265014	CCT5	-0.741825
CCTTAAA**AATAAA**CTTGAGCTGGAATTTGAAAAAAA	chr14	52775193	52775221	GNPNAT1	-0.737359
AA**AATAAA**AAGCCTCAGTGACCCATGAGAAAAAAA	chr1	8861002	8861030	ENO1	-0.728072
TTCTAGGAA**AATAAA**ACCCGTTGATTACTAAAAAA	chr1	156742109	156742137	HDGF	-0.712547
CACA**AATAAA**CAGTGAATTCTCGTTTAAAAA	chrY	2866927	2866951	RPS4Y1	0.703734
GGTC**ATTAAA**ATTACATTGAGGTTCCTACAAAAAAA	chr17	78971253	78971281	LGALS3BP	0.695788

CCTC**AATAAA**GTTTGTGTTTATGCCACCTGAAAAAAAA	chr8	144789765	144789793	RPL8	-0.690137
TC**AATAAA**ACTGTCTTGTGAGCTGAAAAAAA	chr8	27597996	27598019	CLU	0.674317
TATTTGTCATA**AATAAA**TAATGAAAACCTAAAAAA	chr20	62388492	62388520	RPS21	-0.647259
AGGTTTCTA**AATAAA**CAACTGGACCATCAAAAAAAA	chr12	57721529	57721557	OS9	0.640827
TTGAGT**AATAAA**CTTGATTGTAGGAAAAAAA	chr21	34143006	34143030	MRPS6	0.631407
GATG**AATAAA**GCTTCCTGTGTTGTGTGATAAAAAAA	chr4	672436	672464	ATP5I	-0.623522
AAGTCATTTC**ATTAAA**GATGAAAACTTTAAAAAAA	chr2	175178144	175178172	ATP5G3	-0.601364 (1)
GCACGTTGC**ATTAAA**CCTCACTGAAACCTGAAAAAAAA	chr19	1598049	1598077	AC005943.2	-0.600143
TCAGAGCCAC**AATAAA**TTCTATTTCACAAAAAA	chr11	65538678	65538704	SCYL1	0.597803
CGCAAGAACATT**AATAAA**CTAAAAACTTCAAAAAAA	chr10	78040669	78040697	RPS24	-0.246656

(1): Differential usage ("polyA DU" events)

Table S 2: Effect of masking on numbers of raw k-mers, DE k-mers and contigs. Here DE-kupl was run on the EMT dataset using DESeq2 mode, using for the masking step either the full Gencode annotation (default mode), a simplified annotation comprising 1 major transcript per gene, or no mask at all.

	Nb k-mers	Test	raw p-value thres. after BH	nb of DE k-mers	% of DE k-mers from Gencode	nb of contigs
Gencode	40,398,848	T test	0.004720	3,813,418	0	133,690
		DE-seq2	0.007552	6,102,447	0	169,613
1 transcript/gene	51,220,879	T test	0.005728	5,867,874	29.86	182,955
		DE-seq2	0.008661	8,872,987	27.68	217,837
No mask	92,525,450	T test	0.01115	20,634,710	73.77	391,757
		DE-seq2	0.014499	26,831,028	72.12	422,866

Table S 3: Effect of masking on event counts. DE-kupl was run on the EMT dataset using DESeq2 mode and two different masking reference transcriptomes: Gencode and 1-transcript/gene. Event annotation rules were the same as described in the main text.

	Gencode		1-transcript/gene	
Event class	contigs	loci	contigs	loci
splice	3000	1826	7016	3748
splice DU	631	535	1933	1447
polyA	178	159	206	184
polyA DU	24	22	28	26
antisense	784	303	843	327
lincRNA	1676	543	1754	571
SNV	12797	4385	13707	4656
SNV DU	1402	930	1689	1098
intron	57839	7055	80776	8415
intron DU	14323	3878	20999	4789
repeat	1660	839	2025	969
split	0	0	0	0
unmapped	355	0	367	0

Table S 4: Recall of IRfinder and KisSplice predictions by DE-kupl. DE-kupl was run on the EMT dataset using DESeq2 mode and the 1-transcript/gene reference. Other DE-kupl parameters and event annotation rules were the same as described in main text. Parameters for IRfinder and KisSplice are provided in Methods.

		Percent of predictions matched by:		
		Dekupl DE events	Dekupl DU event P<0.05	Dekupl DU event P<0.01
IRfinder	Total predictions (319)	77	68	62
	Top 100 predictions	86	80	74
KisSplice	Total predictions (3616)	36.4	29	24.2
	Top 100 predictions	85	82	82

Table S 5: Contig annotation table
Information is extracted from alignments and overlap with annotations.

	TERM	TYPE	COMMENT
Info	ID	character	contig ID (representative k-mer)
	LineInSam	integer	line number in Gsnap SAM file
Alignment	is_mapped	boolean	True if contig is mapped
	nb_hit	integer[1]	number of aligned positions in the SAM/BAM
	nb_mismatch	integer[1]	mismatch number
	nb_deletion	integer[1]	deletion number
	clipped_5p	integer[1]	clipped bases from 5'
	clipped_3p	integer[1]	clipped bases from 3'
	aligner	character[1]	GSNAP, Blast
Locus	chromosome	character[1]	chromosome of contig
	start	integer[1]	start of contig on chromosome
	end	integer[1]	end of contig on chromosome
	strand	character[1]	strand of contig on chromosome
	gene	character[1,2]	overlapping gene (ENSEMBL ID) on same strand.
	HUGO_ID_gene	character[1,2]	overlapping gene (HUGO ID) on same strand.
	as_gene	character[1,2]	overlapping antisense gene (ENSEMBL ID).
	HUGO_ID_as_gene	character[1,2]	overlapping antisense gene (HUGO ID).
	gene_5p	character[1,2]	nearest 5' gene ID (same strand).
	gene_5p_dist	integer[1,2]	nearest 5' gene dist.
	gene_3p	character[1,2]	Nearest 3' gene ID (same strand).
	gene_3p_dist	integer[1,2]	Nearest 3' gene dist.
Event	exon_coord	character[1]	coordinates of exon(s) matched by contig
	UTR	boolean[1]	contig overlaps UTR
	exonic	boolean[1]	contig overlaps exon
	intronic	boolean[1]	contig overlaps intron
	junction	character[1,2]	coordinate of junction(s).
	nb_junction	integer[1]	number of junctions
	other_split	boolean[1]	contig split in non-canonical fashion (head-to-head, trans-splice, fusion)
	SNV	boolean[1]	contig contains SNV
	gene_is_diff	boolean[1]	gene to which contig is mapped is differentially expressed
	DU_Pvalue	float[1]	P value for contig differential usage
	DU_stat	float[1]	differential usage statistic

[1]: value is NA when contig is not mapped.
[2]: value is "none" when contig does not match a gene.

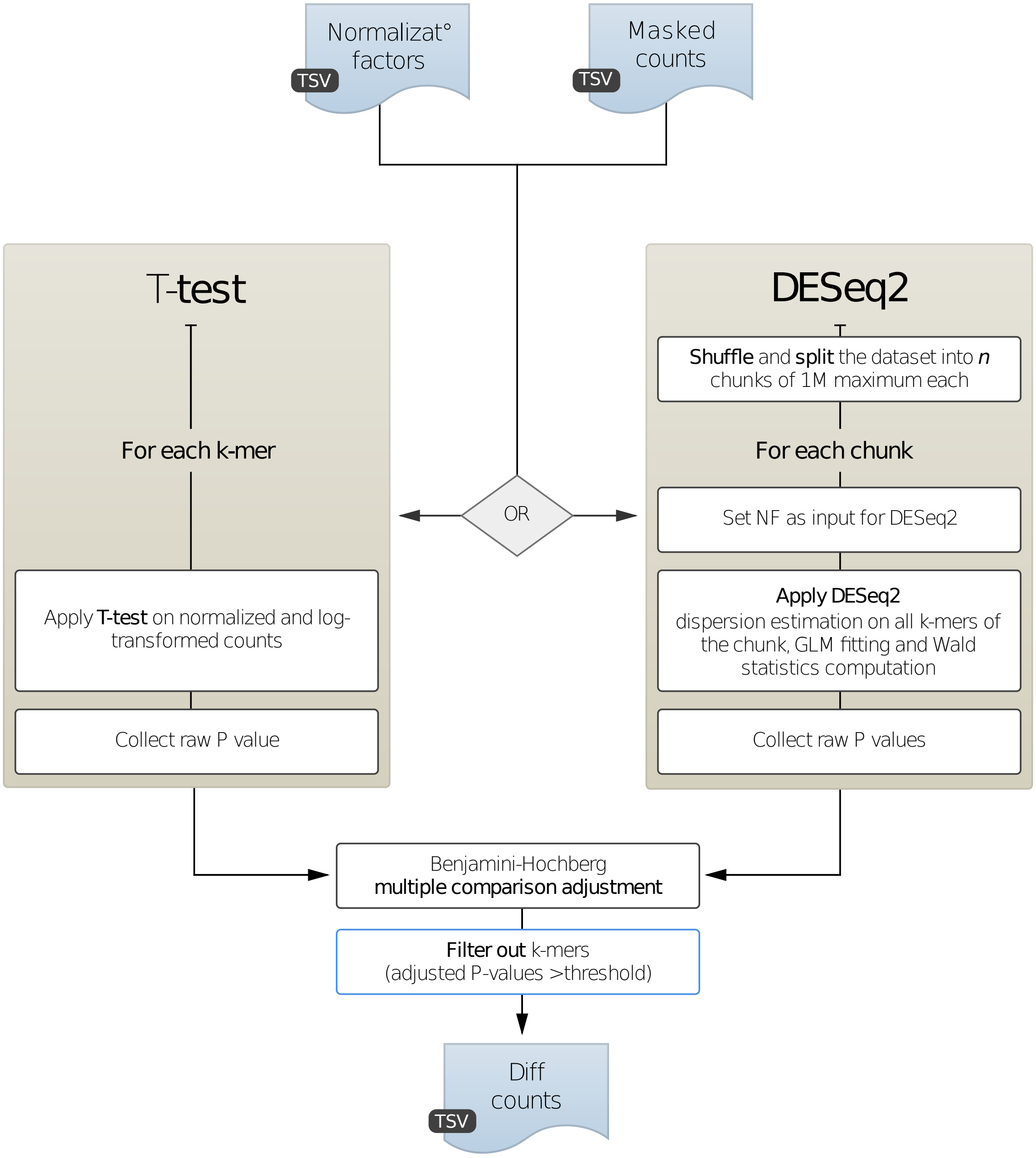

Figure S 1: Differential analysis of k-mers.
Two options are implemented. The first option is to apply a t-test for each k-mer on the log transformed counts, normalized with the previously computed NF. The second option is based on generalized linear model, implemented in the R package `DESeq2` (Love *et al.*, 2014). The first option is implemented in C and run quickly. The second option is slower but increases the ability to detect differential k-mers.

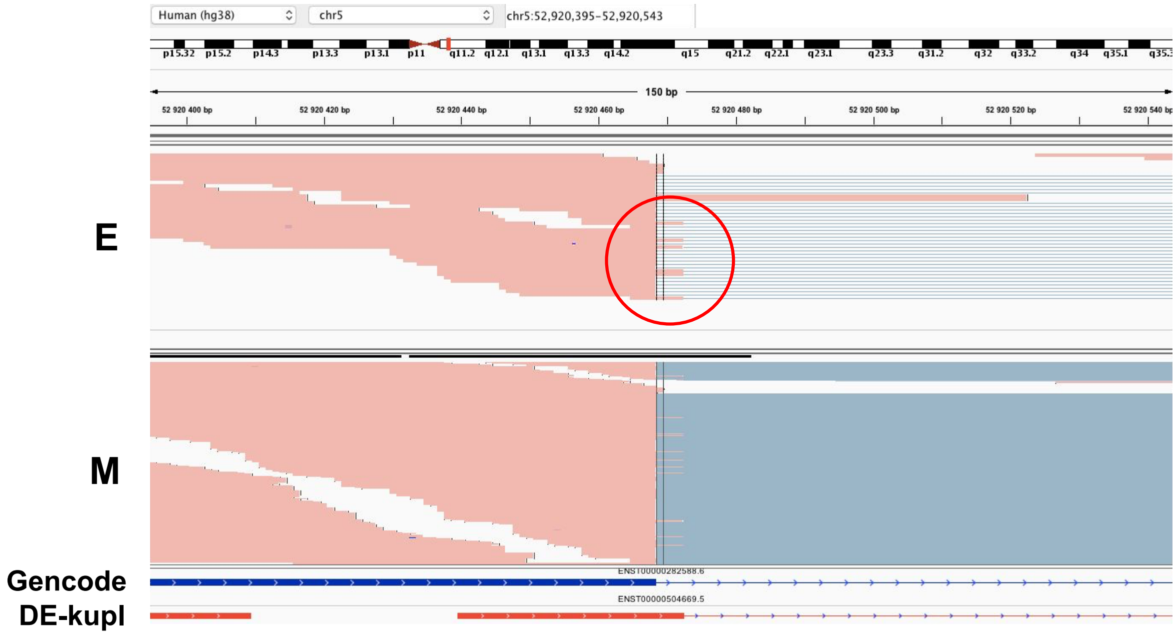

Figure S 2: A novel splicing variant: a 4nt extension in the left exon was not annotated in Gencode and results in a novel DE-kupl contig (circle).
Tracks from top to botttom: chromosome location, RNA-seq reads from 1 Epithelial (E) library, RNA-seq reads from 1 Mesenchymal (M) library, Gencode V.24 annotation, DE-Kupl contigs. This splice variant has a canonical 5'-GT intronic sequence.

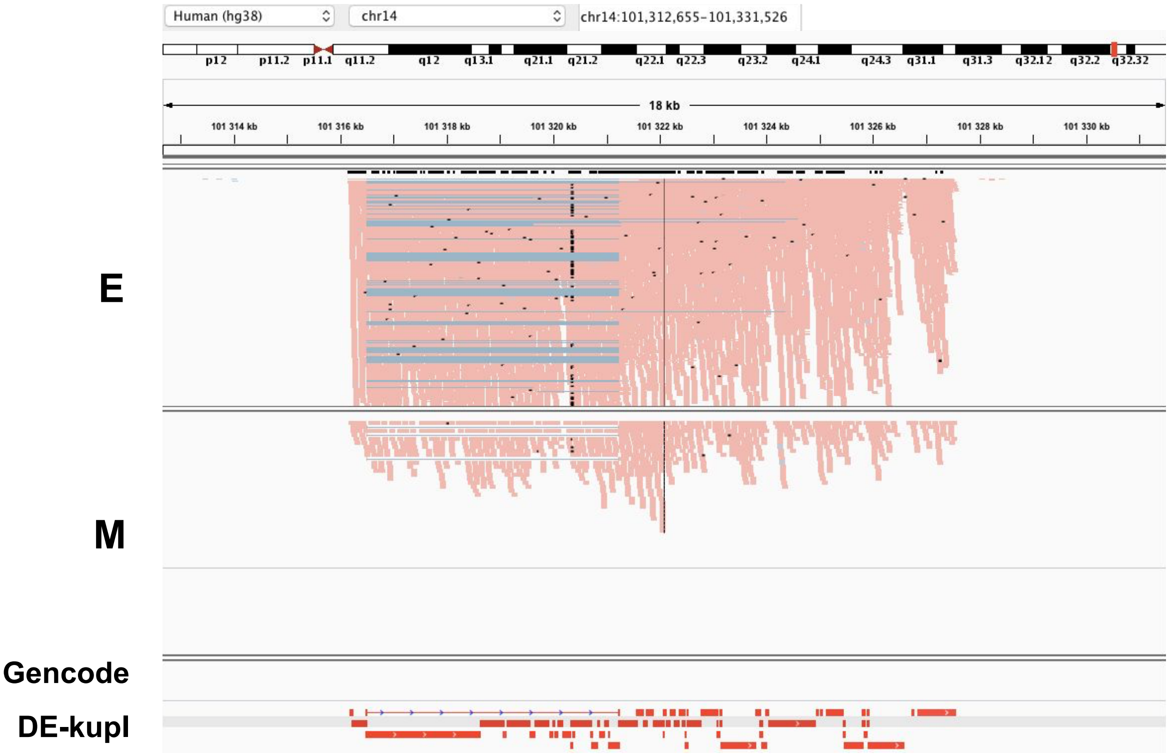

Figure S 3: A novel lncRNA.
See Fig. S2 for legend.

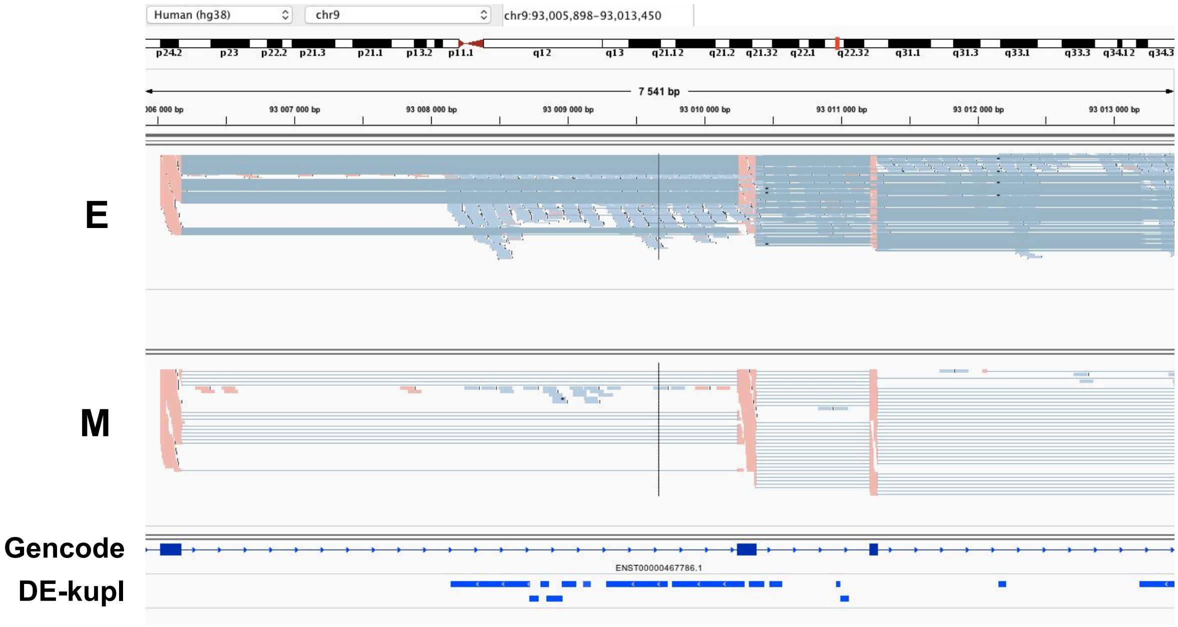

Figure S 4: Antisense transcript associated to potential repression of the sense gene.
See Fig. S2 for legend.

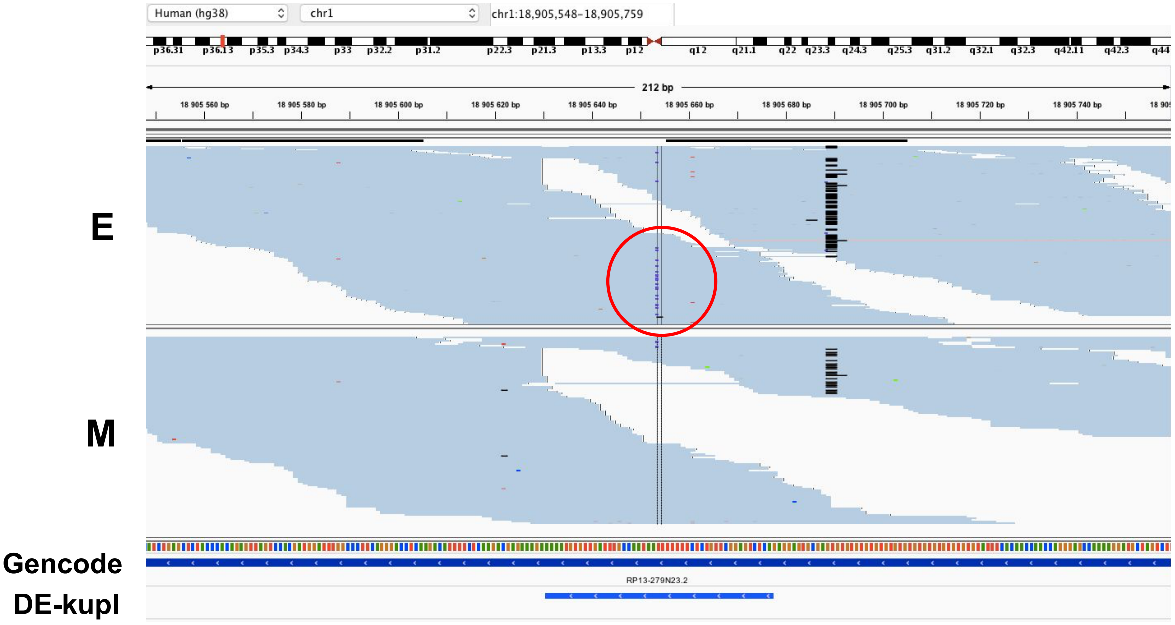

Figure S 5: Potential allele-specific expression.
See Fig. S2 for legend. The central position(circle) has an predominant insertion in condition “E” while the allele is predominantly wild type in condition “M”.

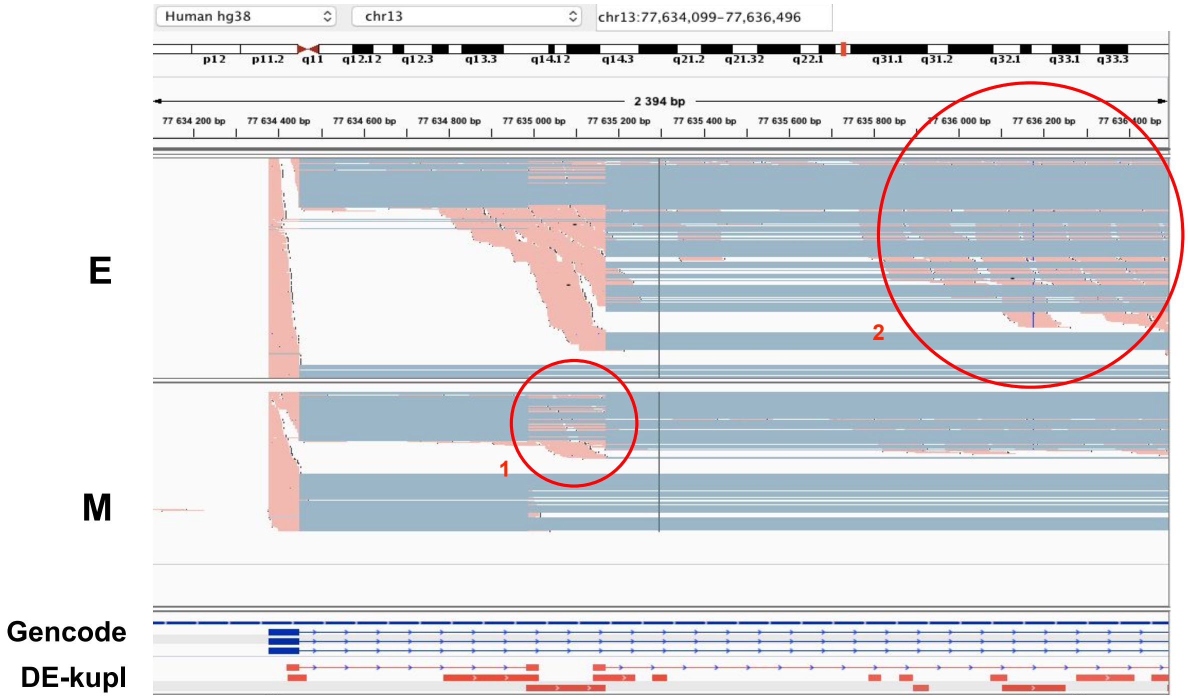

Figure S 6: A combination of differential skipped exon (circle 1) and intron retention (circle 2). See Fig. S2 for legend. The skipped exon is absent in the Gencode annotation.

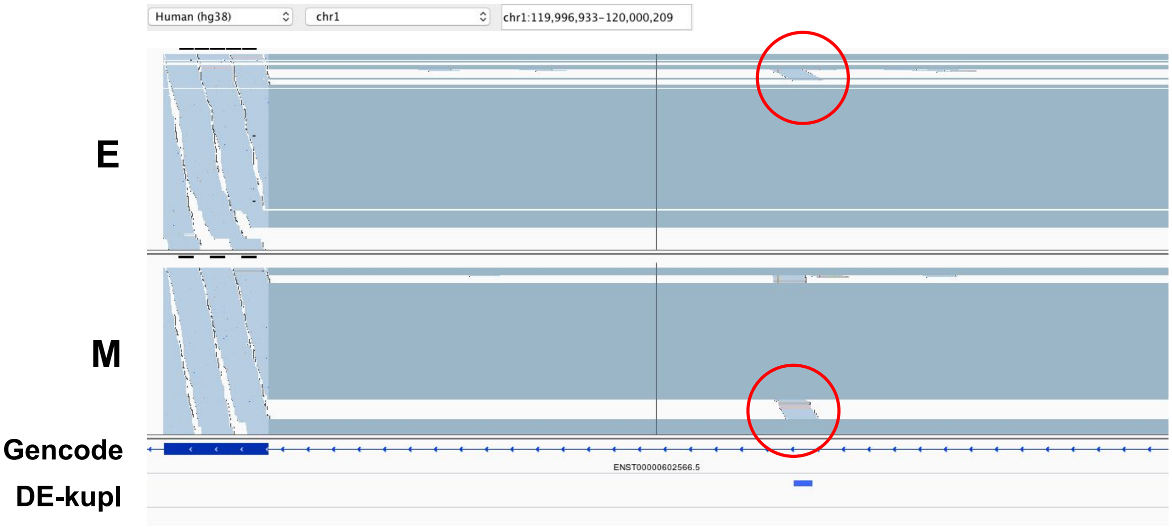

Figure S 7: A case of local intron retention (circle) suggesting RNA processing. See Fig. S 2 for legend.

```
==================================================
file name: RM2_rep.fa_1507579200
sequences:          1136
total length:      86363 bp  (86363 bp excl N/X-runs)
GC level:         45.32 %
bases masked:      53154 bp ( 61.55 %)
==================================================
               number of      length   percentage
               elements*    occupied  of sequence
--------------------------------------------------
SINEs:              136         9399 bp   10.88 %
      ALUs          131         9049 bp   10.48 %
      MIRs            5          350 bp    0.41 %

LINEs:              340        24767 bp   28.68 %
      LINE1         338        24586 bp   28.47 %
      LINE2           1          111 bp    0.13 %
      L3/CR1          1           70 bp    0.08 %

LTR elements:       179        12876 bp   14.91 %
      ERVL           49         4083 bp    4.73 %
      ERVL-MaLRs     24         1849 bp    2.14 %
      ERV_classI     68         4232 bp    4.90 %
      ERV_classII    38         2712 bp    3.14 %

DNA elements:        11          685 bp    0.79 %
     hAT-Charlie      5          255 bp    0.30 %
     TcMar-Tigger     6          430 bp    0.50 %

Unclassified:        27         1971 bp    2.28 %

Total interspersed repeats:    49698 bp   57.55 %


Small RNA:            0            0 bp    0.00 %

Satellites:          33         2339 bp    2.71 %
Simple repeats:      20          889 bp    1.03 %
Low complexity:       4          228 bp    0.26 %
==================================================

* most repeats fragmented by insertions or deletions
  have been counted as one element


The query species was assumed to be homo sapiens
RepeatMasker version open-4.0.6 , default mode

run with rmblastn version 2.6.0+
RepBase Update 20160829, RM database version 20160829
```

Figure S 8: RepeatMasker results for 1136 Contigs with multiple hits in the human genome.

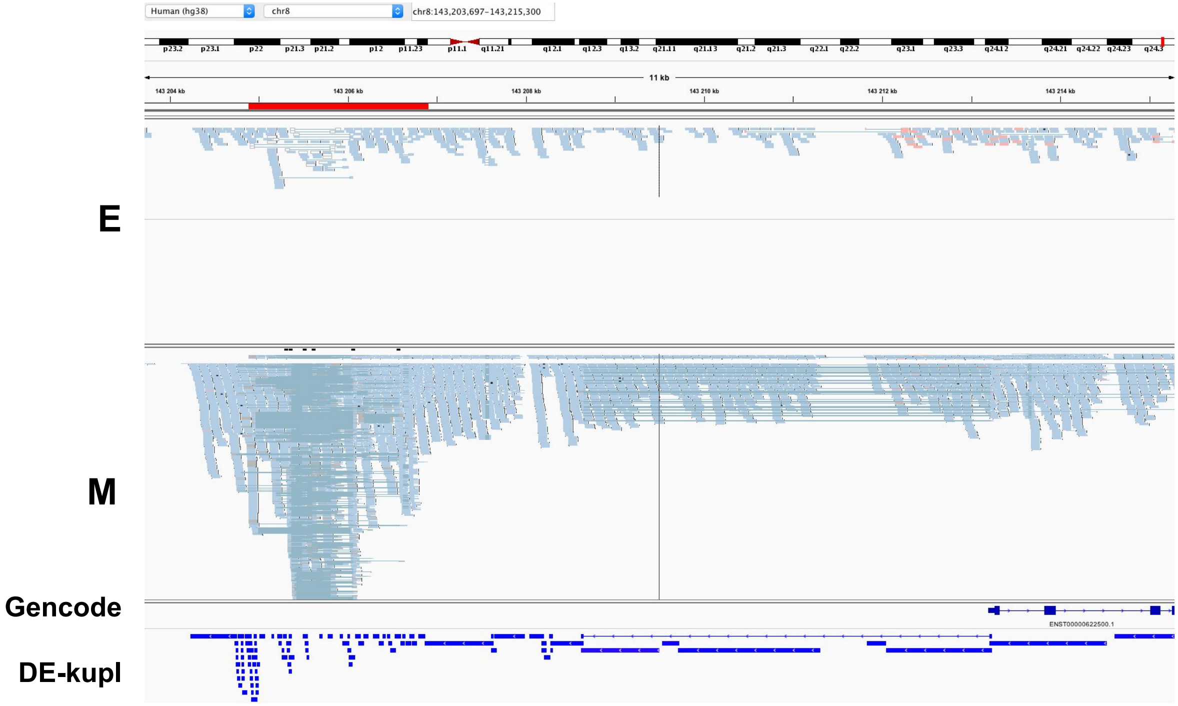

Figure S 9: Tandem repeat at chr8:143,204-870-143,206,916 (red region on top) that is overexpressed in M (bottom) *vs.* E (top) condition.

See Fig. S2 for legend. Note that the overexpressed tandem repeat is part of a larger overexpressed unannotated locus.

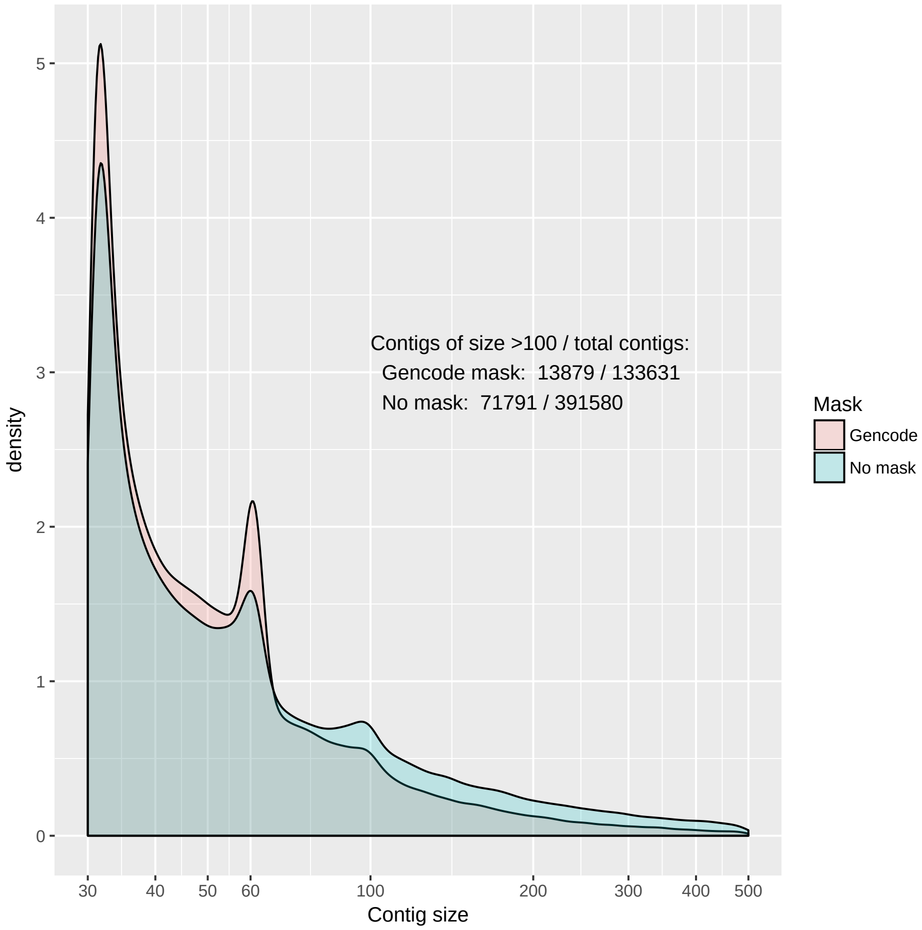

Figure S 10: Effect of masking on contig size.
The density distribution of contig sizes is shown for two DE-kupl runs performed using t-test mode, using Gencode masking or no masking. Numbers of contigs larger than 100nt and total nunbers of contigs are shown for each run.

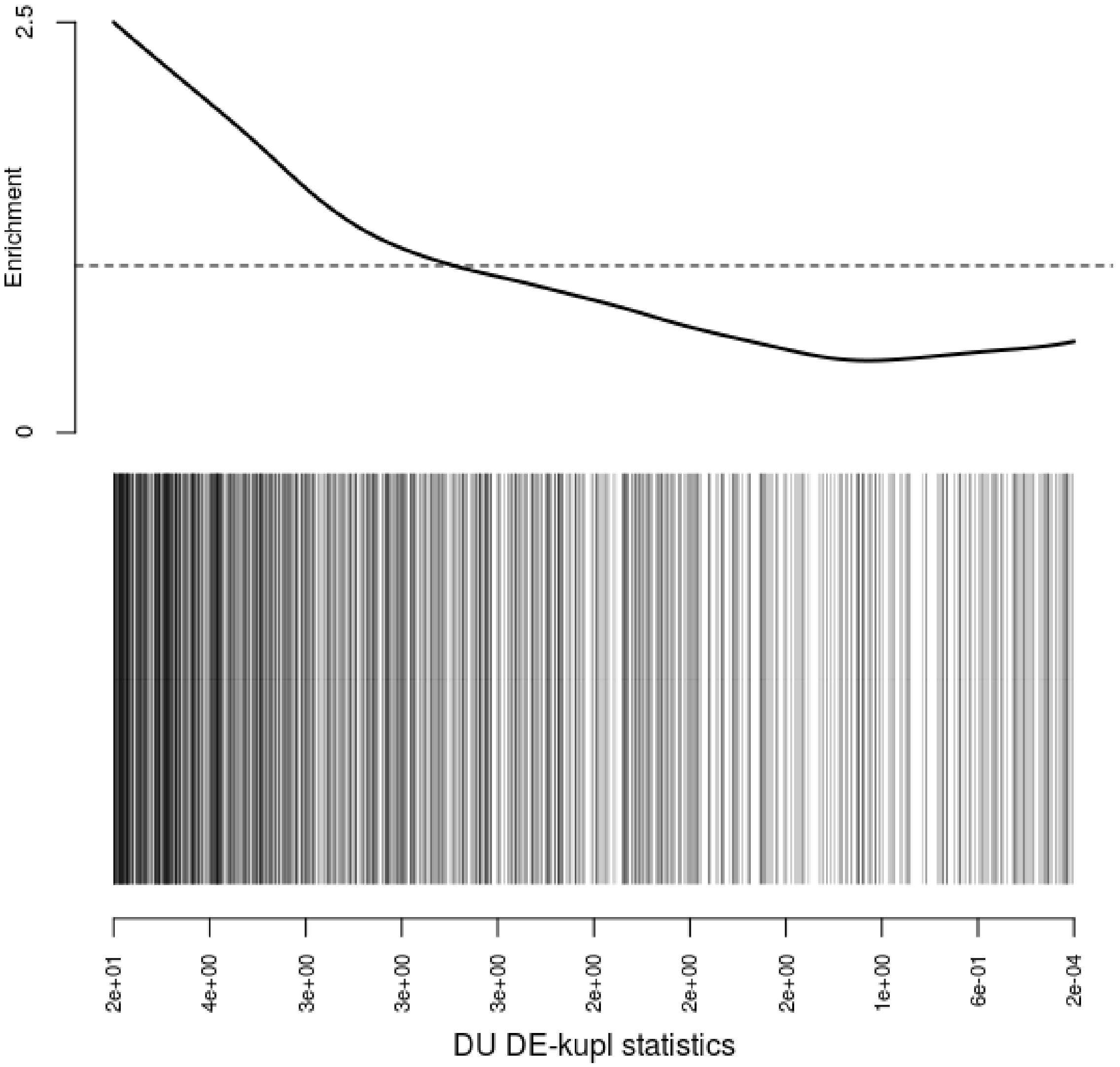

Figure S 11: Barcode plot showing the enrichment of contigs associated with events detected by IRFinder among the ranked list of intronic contigs detected by DE-kupl.
DE-kupl was run using the Deseq2 mode. Masking was done using the one transcript per gene reference. Among the 217,837 DE contigs, 80,776 correspond to intronic events. The 80,776 contigs are tested for DU using the R package `DESeq2` (taking into account the overall expression of genes). Contigs are ranked by statistics in absolute value, from the most significant to the less significant differential contigs (x-axis). IRFinder detects 319 intron retention events, among which 246 events are detected by DE-kupl (77%). Contigs matching events found by IRFinder are marked by vertical bars, forming a barcode-like pattern. An enrichment curve shows the relative enrichment of the vertical bars across the plot. Most of the contigs associated with IRFinder events rank at the top of the ranked list of intronic contigs detected by DE-kupl. The graphic was plotted using a slightly modified version of the `barcode` function of the R package `limma` (Gordon K. Smyth,2004).

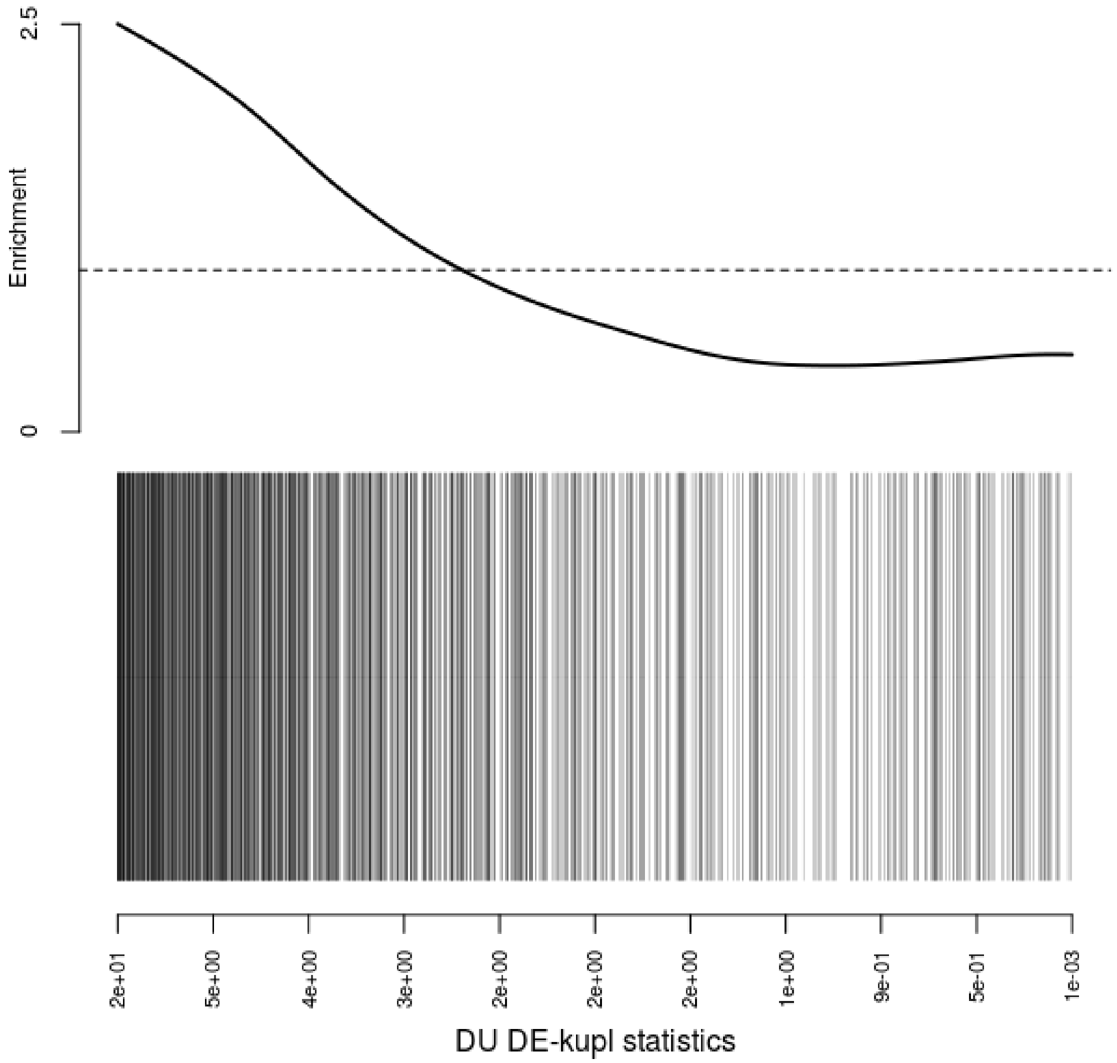

Figure S 12: Barcode plot showing the enrichment of contigs associated with events detected by KisSplice among the ranked list of splice contigs detected by DE-kupl.
DE-kupl was run using the Deseq2 mode. Masking was done using the one transcript per gene reference. Among the 217,837 DE contigs, 7,016 correspond to splice events. The 7,016 contigs are tested for DU using the R package `DESeq2` (taking into account the overall expression of genes). Contigs are ranked by statistics in absolute value, from the most significant to the less significant differential contigs (x-axis). KisSplice predicted 3,616 cases of differential splicing. Among these, 1315 match a DEkupl contig (36%). Among the top 100 KisSplice events (lowest P-value), 85 match a DEkupl contig annotated as splice DU. Contigs matching events found by KisSplice are marked by vertical bars, forming a barcode-like pattern. An enrichment curve shows the relative enrichment of the vertical bars across the plot. Most of the contigs associated with KisSplice events rank at the top of the ranked list of splice contigs detected by DE-kupl. The graphic was plotted using a slightly modified version of the `barcode` function of the R package `limma` (Gordon K. Smyth,2004).

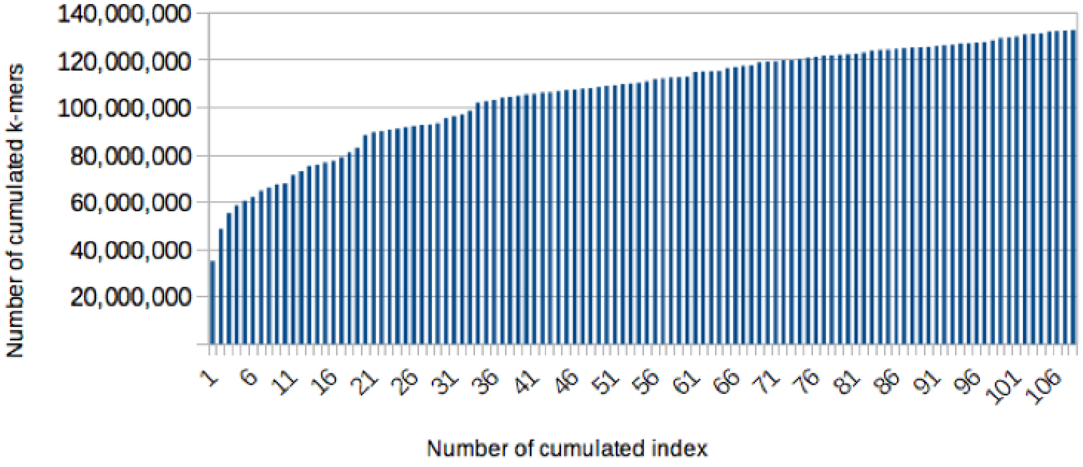

Figure S 13: Growth of cumulative k-mer count with library number.
Here 106 RNA-seq libraries from TCGA (prostate cancer) were indexed and unique 31-nt k-mers were counted using the filtering criterion that a k-mer must be found at least 10 times in one of the libraries.

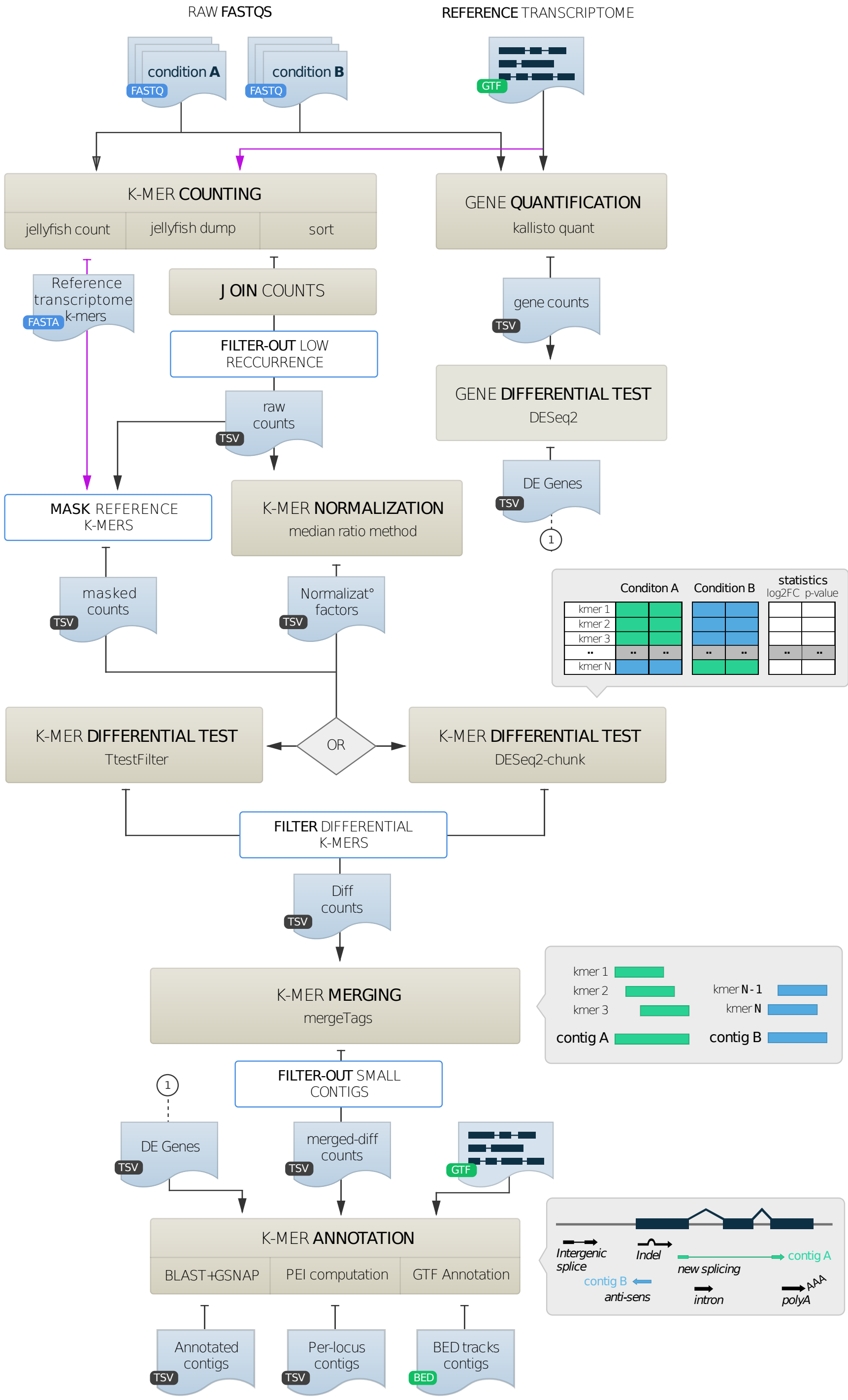

Figure S 14: Detailed view of the DE-kupl pipeline.
First, Jellyfish is applied to count k-mers in all libraries. K-mers counts are then joined into a count matrix and filtered for low-recurrence and matching to the reference transcriptome. Normalization factors are computed from raw K-mer counts and the DE procedure is applied. Finally overlapping DE k-mers are merged into contigs and annotated based on their alignment to reference and overlap with annotations. In parallel, FASTQs are processed with Kallisto to estimate gene-level counts and differentially expressed genes are derived using DESeq2. The list of DE genes is used for contig annotation only.

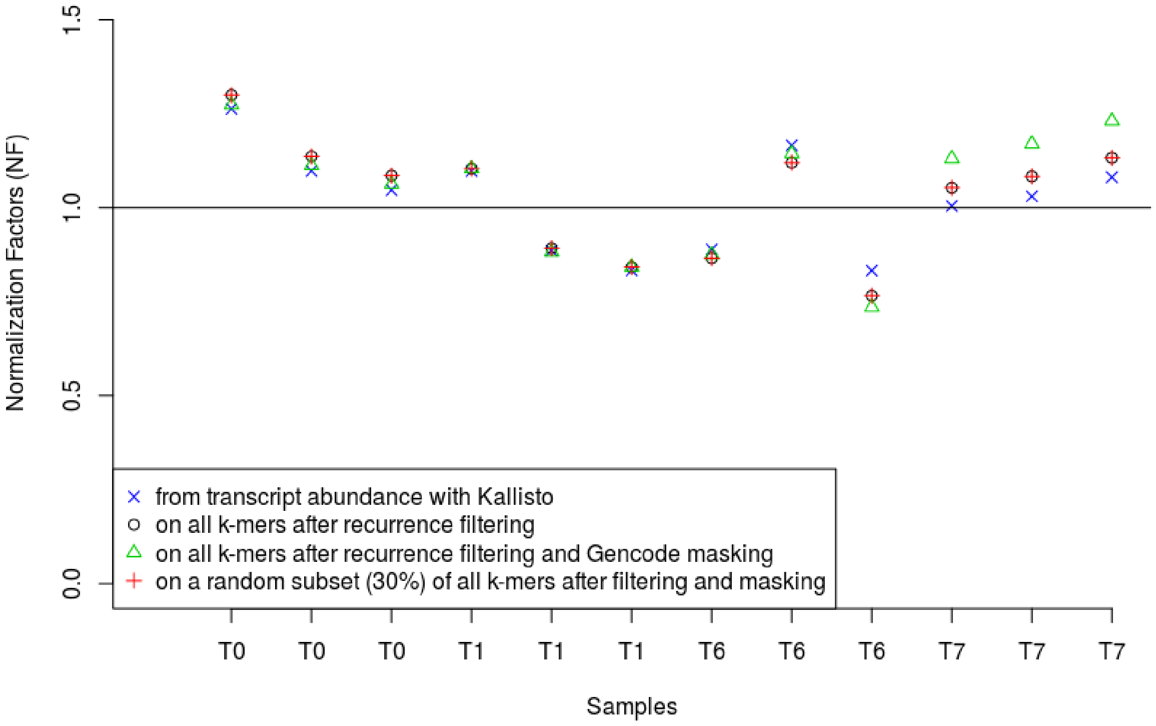

Figure S 15: Normalization factors (NF) computed for the EMT experiment.
All NF are computed using the *median ratio method* (Anders *et al.*, 2010). Blue NF are computed on the table of transcripts abundance obtained on RNA-seq processed with kallisto (conventional pipeline). Black NF are computed on the table of k-mers counts after recurrence filter. Green NF are computed on the table of k-mers counts after recurrence filtering and Gencode masking. Red NF are computed on a subtable aleatory extracted from the table of k-mers after recurrence filter.

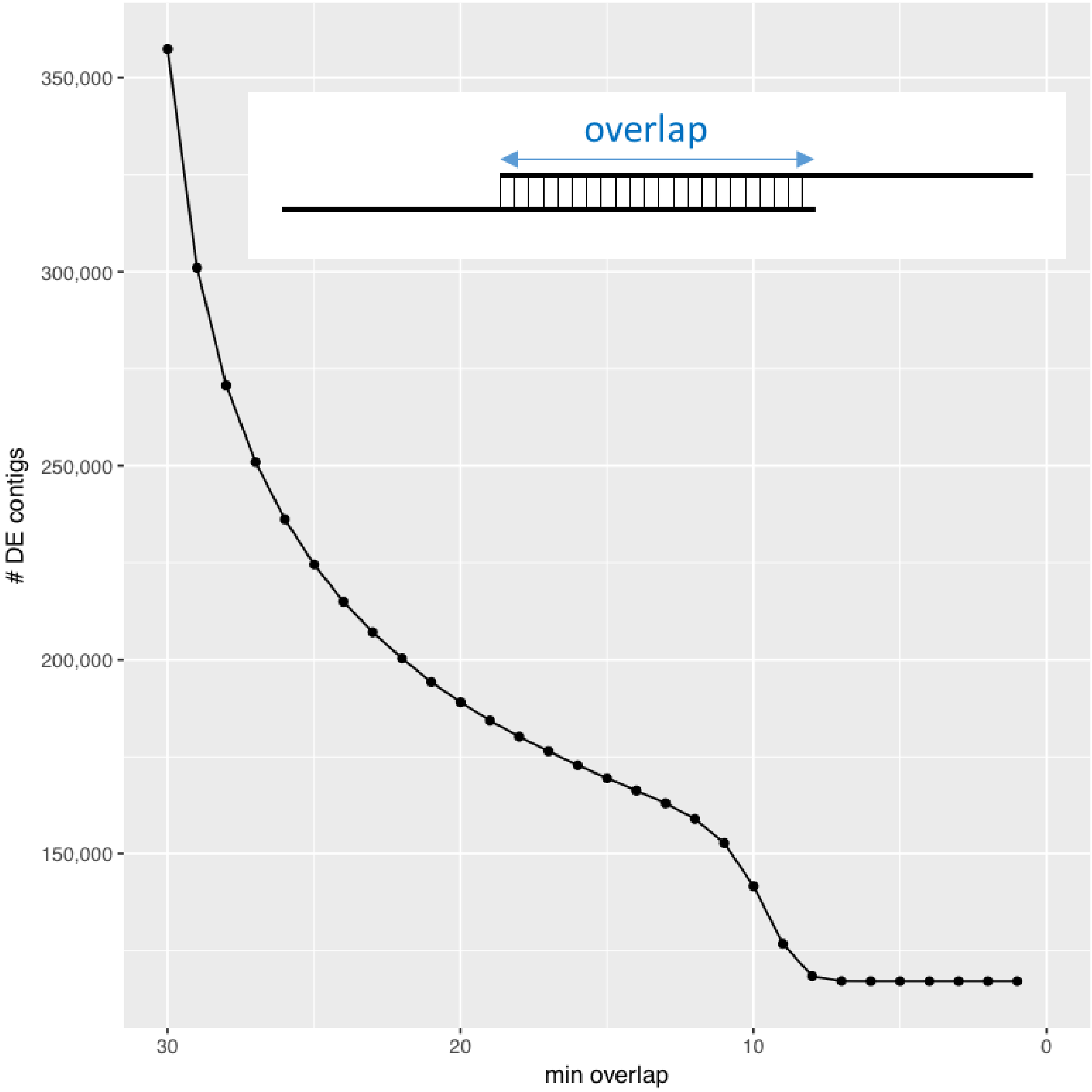

Figure S 16: Effect of minimal overlap on contigs number.
In the k-mer extension procedure, DE k-mers are iteratively extended with overlapping DE k-mers, considering a perfect overlap of k-1, k-2, ..., k-i, where ”k-i” is the minimal overlap allowed. Here k-mer extension was performed with minimal overlaps ranging from 30 to 1. Initial k-mers (n=6,102,447) were obtained from running DE-kupl on the EMT dataset with parameters test=DESeq2, masking: Gencode.
